# From cognitive screening to digital phenotyping: rethinking early detection of cognitive impairment in primary care

**DOI:** 10.3389/fneur.2026.1870463

**Published:** 2026-07-07

**Authors:** Miren Altuna

**Affiliations:** 1 Center for Research and Memory Clinic, CITA-Alzheimer Foundation, Donostia-San Sebastián, Spain; 2 Department of Neurology, Bioaraba Health Research Institute, Araba University Hospital-Txagorritxu, Vitoria-Gasteiz, Spain; 3 Department of Medicine, Faculty of Health Sciences, University of Deusto, Bilbao, Spain

**Keywords:** Alzheimer’s disease, artificial intelligence, cognitive screening, digital biomarkers, mild cognitive impairment, primary care, speech analysis

## Abstract

Early detection of cognitive impairment has become a clinical and public-health priority in the therapeutic era of Alzheimer’s disease (AD). Brief instruments such as the Mini-Mental State Examination, Montreal Cognitive Assessment, Mini-Cog, Clock Drawing Test, Fototest, Memory Alteration Test, Eurotest, and AD8 remain widely used because they are inexpensive, clinically interpretable, and feasible in routine care. However, most were developed to identify established cognitive impairment rather than subtle mild cognitive impairment or biologically defined early AD. Ceiling effects, educational and cultural bias, examiner variability, and limited integration with functional, neuropsychiatric, subjective, and biomarker data restrict their value as stand-alone tools in contemporary diagnostic pathways. Digital cognitive assessment may address selected limitations of conventional screening by standardizing administration, reducing scoring variability, enabling repeated measurement, and capturing process-level features such as response latency, intra-individual variability, learning effects, speech and language markers, graphomotor dynamics, gaze, and ecologically sampled behavior. These signals may support earlier risk stratification and longitudinal monitoring, but they do not resolve diagnostic uncertainty or establish AD etiology on their own. This narrative review examines the transition from traditional cognitive screening to digital cognitive phenotyping. It considers established brief and contextual instruments, digitized conventional tests, remote repeated assessments, speech and language-derived digital cognitive biomarkers, digital clock drawing, prospective-memory tools, virtual reality and serious games, oculomotor and graphomotor metrics, passive sensing, multimodal platforms, and their integration with structural MRI, blood-based biomarkers, cerebrospinal fluid markers, and amyloid/tau PET. In the context of evolving AD criteria and disease-modifying therapies, digital screening should be understood as a governed triage and phenotyping layer rather than a stand-alone diagnostic label. A staged pathway is proposed that combines analog cognitive instruments, informant and functional measures, neuropsychiatric assessment, digital signals, and biological markers to support earlier, more equitable, and clinically actionable detection of cognitive impairment.

## 1 Introduction and rationale: cognitive screening after the therapeutic turn in Alzheimer’s disease

Dementia has become one of the most pressing public-health challenges of aging societies. In 2021, approximately 57 million people worldwide were living with dementia, with nearly 10 million new cases every year; Alzheimer’s disease (AD) is the most common cause and may account for 60–70% of cases (1–4). Dementia is also one of the major causes of disability and dependency among older people, with substantial societal costs: global dementia-related costs were estimated at US$1.31 trillion in 2019 (4). In Spain, AD affects more than 700,000 individuals, with approximately 40,000 new cases diagnosed annually, yet early-stage AD dementia remains underdiagnosed and evidence on mild cognitive impairment (MCI) due to AD is limited (1).

For decades, the main purpose of early detection was to explain symptoms promptly, exclude reversible contributors, plan care, and support patients and caregivers. That purpose has now expanded. The approval of anti-amyloid disease-modifying therapies (DMT), together with the greater availability of fluid and imaging biomarkers, has moved cognitive screening closer to the beginning of a diagnostic and therapeutic pathway. Screening is no longer only a practical method for identifying established dementia; it is increasingly the first step toward etiological confirmation, disease staging, treatment-readiness assessment, clinical-trial referral, or longitudinal monitoring. The phase 3 trials of lecanemab and donanemab in early AD illustrate this shift, as both therapies were studied in early symptomatic populations and required biological evidence of AD pathology (5, 6).

Primary care is central to this pathway. Most people with early cognitive symptoms do not first present to specialist memory clinics with a clear dementia syndrome. They present with memory concerns, subtle changes in daily functioning, mood or sleep symptoms, reduced confidence in complex tasks, or concerns raised by relatives. These early presentations are easy to miss. High educational attainment and cognitive reserve may further mask impairment on brief tests, whereas low educational attainment may produce low scores that are difficult to interpret. The clinical challenge is therefore not simply to detect dementia, but to identify meaningful cognitive change early enough to inform prognosis, follow-up, biomarker testing, and therapeutic planning.

Brief cognitive instruments have long served as the entry point to this process. The Mini-Mental State Examination (MMSE) (7, 8), Montreal Cognitive Assessment (MoCA) (9), Mini-Cog (10), Clock Drawing Test (11), Addenbrooke’s Cognitive Examination, Memory Alteration Test (M@T) (12), Fototest (13), Eurotest, Memory Impairment Screen, and informant measures such as the AD8 (14) are familiar, inexpensive, and feasible in routine care. Their continued use is justified: they provide a common clinical language, support rapid triage, and offer interpretable evidence of objective cognitive performance or observed change.

Cognitive scores alone are not enough. Early cognitive impairment becomes clinically meaningful when interpreted alongside subjective concern, informant-reported change, functional status, neuropsychiatric symptoms, medical comorbidity, and medication exposure. Informant tools such as AD8 or IQCODE help determine whether current performance represents decline from a previous level (14). Functional measures such as the Interview for Deterioration in Daily Living Activities in Dementia, Functional Activities Questionnaire, Lawton–Brody Instrumental Activities of Daily Living scale, Barthel Index, and Katz Index help distinguish MCI from dementia by establishing whether cognitive change affects everyday independence. Neuropsychiatric instruments such as the Neuropsychiatric Inventory or NPI-Q capture symptoms that may precede, accompany, mimic, or worsen cognitive impairment, including apathy, depression, anxiety, irritability, psychosis, sleep disturbance, and disinhibition (15, 16). Table 1 summarizes these clinical information domains and the main interpretive limitations that arise when each layer is used in isolation.

**Table 1:** Clinical information domains relevant to early screening of cognitive impairment.

Clinical domain	Representative instruments/examples	Clinical contribution	Main limitation when used alone
Patient-reported cognitive change	Cognitive Concerns Index; Cognitive Complaint Interview; subjective cognitive decline (SCD) scales; structured memory-complaint questionnaires	Captures the individual’s perception of decline and may identify help-seeking patients before objective impairment is demonstrable on brief testing.	Poor specificity; self-report is strongly influenced by depression, anxiety, sleep disturbance, personality traits, and health beliefs.
Informant-reported change	AD8; IQCODE; structured caregiver interview	Provides evidence of decline from prior baseline and helps contextualize current performance, particularly when cognitive reserve or low education complicates interpretation of test scores.	Depends on the availability and reliability of an informant; reports may be shaped by caregiver burden, emotional distress, or limited day-to-day observation.
Brief cognitive performance	MMSE; MoCA; Mini-Cog; Clock Drawing Test; M@T; Fototest; Eurotest; MIS	Offers a rapid, structured estimate of current cognitive performance and supports first-line triage in primary care and specialist settings.	Limited etiological specificity; performance is influenced by education, culture, language, and sensory factors, and some instruments show ceiling effects in early disease.
Functional status	IDDD; FAQ; Lawton–Brody IADL scale; Barthel Index; Katz Index; Disability Assessment for Dementia	Establishes whether cognitive change has consequences for daily life and helps distinguish MCI from dementia.	Functional impairment is not specific to cognitive disorders and may also reflect frailty, sensory loss, motor disability, environmental demands, or caregiver compensation.
Neuropsychiatric and affective profile	NPI; NPI-Q; GDS; PHQ-9; anxiety scales; sleep questionnaires	Identifies behavioral symptoms, affective comorbidity, and potentially reversible contributors to poor cognitive performance.	These symptoms are clinically important but not specific to AD; they may precede, accompany, mimic, or exacerbate cognitive impairment.
Digital cognitive phenotype	Speech and language analysis; remote memory tasks; digital clock drawing; reaction-time paradigms; graphomotor metrics; eye tracking; virtual reality tasks; wearable-derived measures	Adds process-level and longitudinal information that conventional testing usually misses, such as latency, intra-individual variability, learning effects, speech pauses, motor behavior, gaze patterns, and ecologically sampled performance.	Requires robust technical, psychometric, clinical, and fairness validation; performance may also be affected by digital literacy, device familiarity, access, and privacy constraints.
Biological and structural confirmation	Plasma p-tau217 / p-tau181; Aβ42/40; GFAP; NfL; structural MRI; hippocampal or medial temporal atrophy; nWBV; CSF biomarkers; amyloid PET; tau PET	Supports etiological clarification, staging, prognostic assessment, and treatment-readiness decisions in the therapeutic era of AD.	Interpretation depends on clinical context; access, cost, assay variability, and the risk of overdiagnosis limit use as stand-alone screening tools.

AD, Alzheimer’s disease; AD8, Ascertain Dementia 8; Aβ, amyloid beta; CSF, cerebrospinal fluid; FAQ, Functional Activities Questionnaire; GDS, Geriatric Depression Scale or Global Deterioration Scale, according to context; GFAP, glial fibrillary acidic protein; IADL, instrumental activities of daily living; IDDD, Interview for Deterioration in Daily Living Activities in Dementia; IQCODE, Informant Questionnaire on Cognitive Decline in the Elderly; M@T, Memory Alteration Test; MCI, mild cognitive impairment; MIS, Memory Impairment Screen; MMSE, Mini-Mental State Examination; MoCA, Montreal Cognitive Assessment; MRI, magnetic resonance imaging; NfL, neurofilament light; NPI, Neuropsychiatric Inventory; PET, positron emission tomography; PHQ-9, Patient Health Questionnaire-9; p-tau, phosphorylated tau; nWBV, normalized whole-brain volume.

In Spain, population-based and cohort data underscore the scale of the issue. The EPIC-Spain Dementia Cohort found that dementia incidence increases with age, is higher in women, and is influenced by lower education levels and vascular risk factors (17). Modeling studies also predict a growing dementia burden in Spain over the next few decades (18). A recent systematic review estimates that over 700,000 people in Spain have AD, with about 40,000 new cases diagnosed each year. It also highlights the underdiagnosis of early-stage AD dementia and the limited evidence on MCI due to AD (1). Frequently reported instruments include the MMSE, Barthel Index, and Global Deterioration Scale, while etiological workup often lacks core AD biomarkers. The review also highlights substantial caregiver burden and a predominance of non-healthcare costs, largely driven by informal care. This pattern suggests that many patients are still identified after functional decline has become established, rather than at stages where biomarker confirmation, clinical-trial access, care planning, and treatment-readiness assessment may be most useful.

Digital cognitive assessment has emerged in response to this gap. Some tools digitize established tests, allowing standardized administration, automated scoring, precise timing, remote access, and reduced examiner variability. Others are native digital instruments designed to capture signals unavailable to paper-and-pencil testing: response latency, intra-individual variability, learning curves, practice effects, graphomotor dynamics, eye movements, speech pauses, lexical diversity, gait, keystroke patterns, and performance in simulated real-world tasks (19–23).

**Figure 1:**
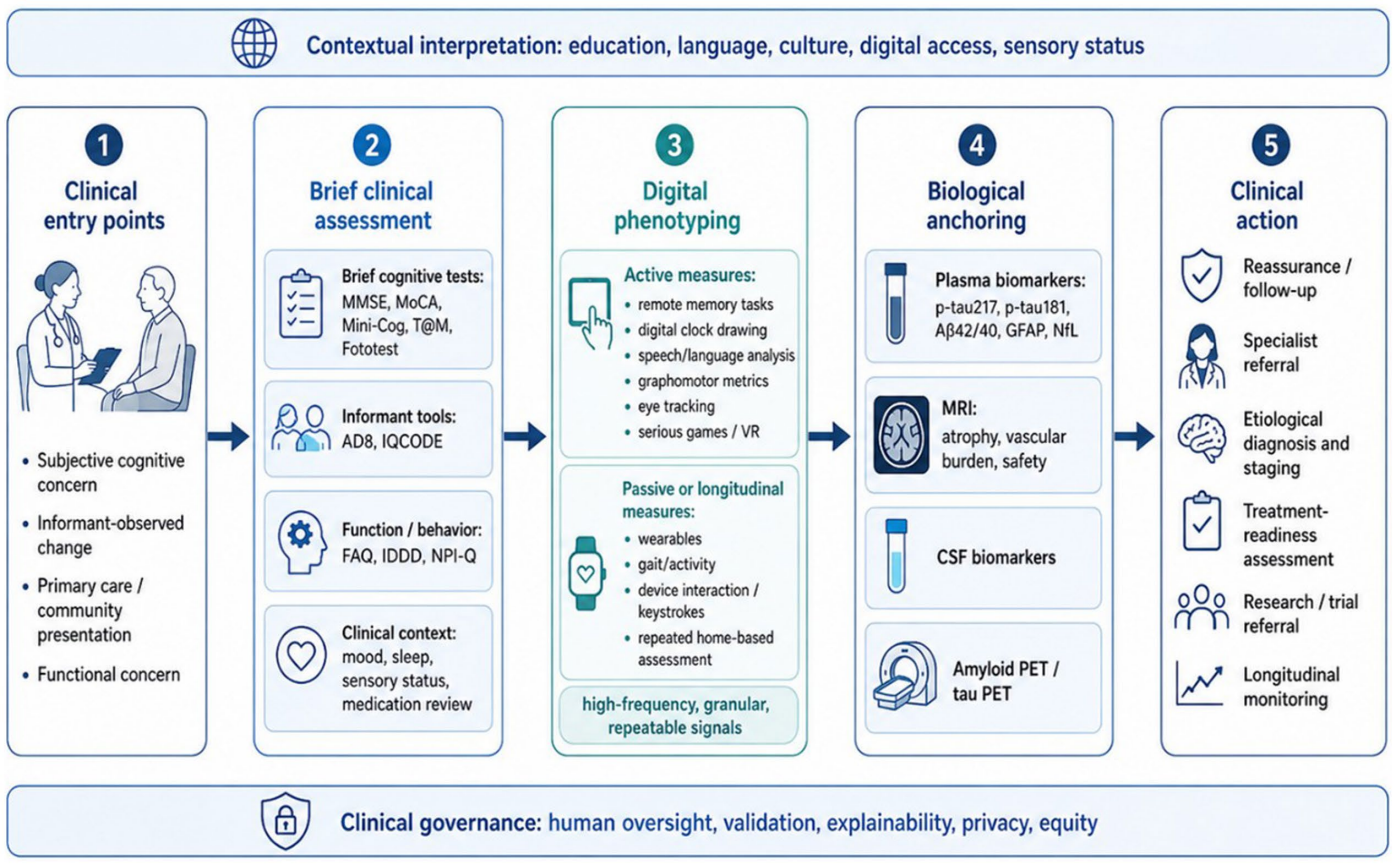
Multimodal pathway for early cognitive impairment screening. The figure created with Biorender.com summarizes a stepwise framework for early cognitive impairment detection, beginning with patient- or informant-reported concern and brief clinical assessment, followed by contextual characterization through functional, neuropsychiatric, and subjective measures. AD8, Ascertain Dementia 8; Aβ, amyloid beta; CSF, cerebrospinal fluid; FAQ, Functional Activities Questionnaire; GFAP, glial fibrillary acidic protein; IDDD, Interview for Deterioration in Daily Living Activities in Dementia; IQCODE, Informant Questionnaire on Cognitive Decline in the Elderly; MMSE, Mini-Mental State Examination; MoCA, Montreal Cognitive Assessment; MRI, magnetic resonance imaging; NfL, neurofilament light; NPI-Q, Neuropsychiatric Inventory Questionnaire; PET, positron emission tomography; p-tau, phosphorylated tau; T@M, Memory Alteration Test; VR, virtual reality. Created with Biorender.com.

Figure 1 outlines the staged framework used in this review.

Terminology holds vital importance in this field. In this review, digital cognitive assessment is used as the umbrella term for technology-enabled evaluation of cognitive abilities, whether through digital versions of traditional tests or native digital tasks. Digital cognitive phenotyping encompasses a broader characterization of patterns across cognitive, functional, behavioral, linguistic, motor, and interaction domains, using both active and passive digital measures. Digital biomarkers refer more specifically to objective, measurable digital signals—such as speech pauses, response times, graphomotor movements, gaze patterns, or mobility features—that may indicate cognitive, functional, behavioral, or biological states. In summary, digital phenotyping involves profiling, while digital biomarkers are individual measurable features within that profile.

The promise of digital assessment should nevertheless be approached with caution. A digital version of a biased task may preserve the same educational, cultural, sensory, or motor biases as its analog predecessor. Conversely, an artificial-intelligence model may detect subtle patterns but remain poorly interpretable, weakly validated outside selected cohorts, or inequitable across language, education, age, sensory impairment, and digital-access strata. The more clinically useful question is how digital tools can complement existing instruments, improve risk stratification, support longitudinal monitoring, and identify individuals who require confirmatory clinical and biological assessment.

This question has become more urgent with the emergence of DMT. Anti-amyloid monoclonal antibodies have shifted attention toward earlier symptomatic stages, particularly MCI and mild dementia due to AD, where treatment eligibility is likely to be considered. Their use requires biological confirmation of AD pathology, safety assessment, MRI monitoring, specialist supervision, and sustainable reimbursement pathways.

The diagnostic boundary itself is also contested. The 2024 Alzheimer’s Association revised criteria emphasize a biological definition of AD (24), whereas the International Working Group argues for a clinical-biological construct (25) in routine practice and cautions against diagnosing cognitively unimpaired biomarker-positive individuals as having AD solely on the basis of biology. This debate directly affects the interpretation of digital screening. If AD is defined primarily by biomarkers, digital tools may contribute to staging, monitoring, or functional phenotyping after biological abnormality is established. In either model, digital tools should not independently assign an etiological diagnosis.

This critical narrative review examines the transition from traditional cognitive screening to digital and AI-enabled cognitive phenotyping. The central argument is that digital cognitive screening should be understood as a governed, interpretable, multimodal layer within a staged pathway for early detection—not as an automated substitute for clinical judgment or biological confirmation.

## 2 Evidence acquisition and narrative synthesis framework

This article was created as a critical narrative review supported by a structured literature search, not as a formal systematic review or meta-analysis. This choice was made because cognitive screening research is clinically and methodologically diverse: studies vary in target populations, disease stages, settings, reference standards, languages, cultural adaptations, technological platforms, supervision levels, biomarker availability, and intended clinical uses.

The goal of the evidence gathering was not to produce a single pooled measure of diagnostic performance. Instead, the aim was to chart the development of the field and critically evaluate how different tools may fit into a staged approach for early detection of cognitive impairment. The synthesis was guided by four practical questions: what signal is being measured, how reliably it is measured, in whom it has been validated, and what clinical decision it can reasonably support.

### 2.1 Search strategy and scope

A structured PubMed search was conducted on 1 March 2026 using two complementary strategies. The first search aimed to capture the broad literature on digital cognitive screening:

“digital” AND “cognitive” AND “screening”

Filters were applied for the last 10 years, free full text, human studies, and English, Spanish, or French language. This search retrieved 621 records with filters and 764 records without filters. From the filtered results, 154 articles were selected for detailed review because they addressed cognitive screening, digital assessment, MCI, dementia, AD, digital biomarkers, remote monitoring, or implementation of digital tools.

The second search focused on brief cognitive screening tools in relation to MCI and AD:

“brief” AND “cognitive” AND “screening” AND (“mild cognitive impairment” OR “Alzheimer”)

Using the same filters, this search retrieved 222 records, or 571 without filters. From the filtered results, 51 articles were selected for detailed review.

The database search was supplemented by manual review of reference lists, recent reviews, Cochrane diagnostic reviews, Spanish validation studies, regulatory documents, and landmark publications on AD diagnostic criteria, DMT, blood-based biomarkers, digital equity, explainable artificial intelligence, and implementation science.

The structured database component of the search was limited to PubMed/MEDLINE. This is a limitation, as relevant studies on digital cognitive assessment, speech processing, AI/ML models, wearable technologies, passive sensing, and human–computer interaction may also be indexed in Embase, PsycINFO, IEEE Xplore, ACM Digital Library, or other technical databases. This limitation was partly mitigated by manual reference checking and targeted inclusion of recent reviews, regulatory documents, and implementation papers. However, because this article was designed as a critical narrative review rather than a formal systematic review or meta-analysis, the search should be interpreted as a structured evidence-mapping strategy rather than an exhaustive retrieval of all digital cognitive technologies.

Records were screened first at the title and abstract level. Articles were retained for detailed review when they provided empirical evidence, validation data, methodological guidance, biomarker associations, implementation findings, or conceptual relevance to the staged pathway proposed in this manuscript. Priority was given to studies involving older adults, MCI, AD, dementia, primary care, community screening, memory-clinic populations, remote assessment, or biomarker-enriched cohorts. Articles were not prioritized when they focused exclusively on cognitive training or rehabilitation without an assessment component, purely technical algorithm development without clinical validation, pediatric or non-neurodegenerative populations, or established moderate-to-severe dementia without implications for screening, staging, or early detection.

### 2.2 Selection and synthesis logic

The selected literature was organized by the type of signal captured and by the level of evidence required for clinical translation. This approach was used because superficially similar tools may differ substantially in their constructs, validation requirements, and clinical roles. A digitized MoCA, a speech-based classifier, a wearable-derived gait marker, and a plasma p-tau217 assay may all contribute to early detection, but they do not measure the same phenomenon and should not be judged by the same evidentiary standard. Table 2 summarizes the domains used to appraise translational readiness, including technical validity, psychometric performance, construct validity, diagnostic accuracy, biomarker and etiological validity, longitudinal sensitivity, clinical relevance, equity, interpretability, implementation feasibility, and regulatory or ethical readiness.

**Table 2:** Proposed domains for appraising whether a digital cognitive screening tool is ready to move from development to clinical use.

Domain	Minimum evidentiary requirement	Preferred metrics or evidence	Main risk if insufficiently addressed
Intended use and target condition	The tool must specify whether it is intended for population screening, primary-care triage, remote monitoring, trial enrichment, biomarker referral, or treatment-readiness support.	Prespecified use case, target population, target condition, clinical setting, decision threshold, and expected downstream action.	Accuracy estimates become difficult to interpret because MCI, dementia, amyloid positivity, AD pathology, and treatment eligibility are not equivalent targets.
Analytical validity	The digital signal must be captured and processed reliably across devices, software versions, sensors, and test environments.	Signal-quality indices, device equivalence, missing-data rates, unusable-recording rates, preprocessing description, software versioning.	Apparent cognitive differences may reflect hardware, software, sensor, or environmental artifacts.
Measurement properties	The score or feature must be sufficiently stable and reproducible for its intended use.	Test–retest reliability, alternate-form reliability, ICC, standard error of measurement, practice effects, learning curves, missing-data behavior.	A measure may discriminate groups cross-sectionally but remain unsuitable for monitoring or individual-level interpretation.
Construct validity	The digital feature must map onto a clinically meaningful cognitive, functional, linguistic, motor, or behavioral construct.	Convergent and divergent validity against neuropsychological domains, informant measures, functional scales, or theoretically related features.	The model may classify cases without a clear understanding of what clinical construct is being measured.
Diagnostic validity	The tool must discriminate clinically defined groups in the population and setting where it is intended to be used.	Sensitivity, specificity, AUC, likelihood ratios, PPV, NPV, calibration, confidence intervals, prespecified thresholds, external validation.	Case–control inflation, spectrum bias, unstable cut-offs, and poor transferability to primary care or community samples.
Biomarker and etiological validity	Digital outputs should be tested, when relevant, against biological or structural markers of AD or neurodegeneration.	Plasma p-tau, Aβ42/40, CSF biomarkers, amyloid/tau PET, MRI atrophy markers, hippocampal or medial temporal atrophy, vascular burden, nWBV.	Digital tools may be validated only against cognitive labels, while claims about AD pathology or treatment relevance remain unsupported.
Longitudinal validity	The tool should detect within-person change and clinically meaningful trajectories over time.	Reliable change indices, slope estimates, responsiveness, conversion to MCI/dementia, functional decline, biomarker progression, repeated-measures models.	Cross-sectional discrimination may not translate into prognostic value or monitoring utility.
Clinical actionability	The output should support a defined clinical action rather than provide an isolated risk score.	Referral yield, change in management, biomarker-test yield, monitoring decisions, treatment-readiness assessment, clinician-facing interpretation.	The tool may increase uncertainty, referrals, or testing without improving decisions.
Equity and measurement invariance	Performance should be examined across demographic, linguistic, sensory, clinical, and digital-access strata.	Subgroup calibration by age, sex/gender, education, language, ethnicity, sensory status, comorbidity, digital literacy, device access, and care setting.	Aggregate accuracy may conceal systematic misclassification in underrepresented or digitally excluded groups.
Interpretability and uncertainty	Clinicians should understand what the result means, what may confound it, and how uncertain it is.	Transparent scoring rules, interpretable features, uncertainty estimates, known confounders, contraindications, explanation of thresholds.	Black-box outputs may be overinterpreted or used beyond their validated purpose.
Workflow and implementation validity	The tool must fit the intended clinical pathway and available resources.	Completion rate, administration time, staff support, training burden, failed assessments, EHR integration, referral consequences, implementation cost.	Efficiency at the test level may shift workload to imaging, biomarkers, memory clinics, or counseling services.
Regulatory and governance readiness	Privacy, consent, accountability, auditability, and medical-device status should be specified before deployment.	Consent model, data-retention policy, cybersecurity, audit trail, SaMD/MDR/FDA status, post-deployment monitoring, model-update plan.	Sensitive cognitive or behavioral data may be collected or acted upon without adequate oversight, accountability, or regulatory clarity.

The table complements accuracy metrics by emphasizing technical, psychometric, biological, clinical, equity, implementation, and governance requirements. AD, Alzheimer’s disease; AUC, area under the curve; CSF, cerebrospinal fluid; EHR, electronic health record; ICC, intraclass correlation coefficient; MCI, mild cognitive impairment; MRI, magnetic resonance imaging; nWBV, normalized whole-brain volume; NPV, negative predictive value; PET, positron emission tomography; PPV, positive predictive value; SaMD, software as a medical device.

### 2.3 Appraisal and translational readiness

Evidence was interpreted narratively using principles derived from diagnostic-accuracy, psychometric, and implementation-science frameworks, including STARD (26), QUADAS-2 (27–29), COSMIN (30), and related reporting standards. Particular attention was given to five recurrent threats to validity: spectrum bias, heterogeneity in reference standards, educational and linguistic bias, implementation bias, and algorithmic opacity.

For each tool or modality, the review considered diagnostic accuracy, psychometric stability, convergent and discriminant validity, biomarker associations, implementation outcomes, and plausible clinical role.

To avoid treating all digital tools as equally mature, evidence was interpreted according to five levels of translational readiness: technical feasibility, usability and acceptability, psychometric validity, diagnostic or biomarker validity, and clinical implementation readiness. Few have reached full implementation readiness, defined by external validation, workflow integration, equity testing, privacy safeguards, interpretable outputs, and evidence that the tool improves referral, monitoring, clinical decision-making, or patient-centered outcomes.

**Figure 2:**
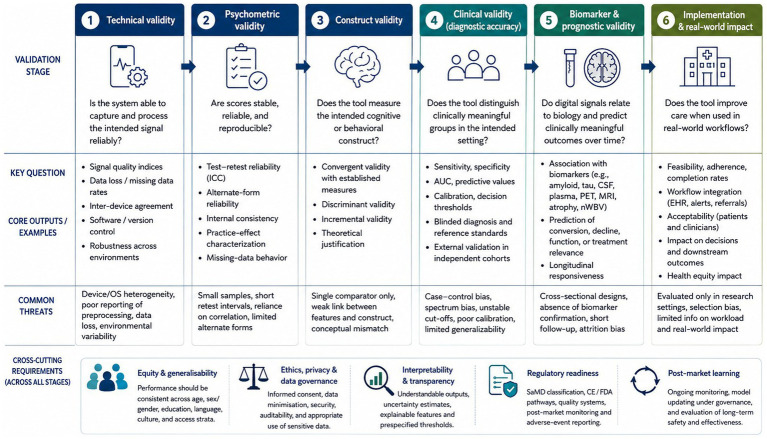
Validation continuum for digital cognitive screening tools. Revised validation framework showing the evidence required to move from signal capture to real-world clinical impact. Technical, psychometric, construct, diagnostic, biomarker/prognostic, and implementation validity should be supported by cross-cutting requirements for equity, governance, interpretability, regulatory readiness, and post-market learning. AD, Alzheimer’s disease; AUC, area under the curve; CE, Conformité Européenne marking; CSF, cerebrospinal fluid; EHR, electronic health record; FDA, U.S. Food and Drug Administration; ICC, intraclass correlation coefficient; MCI, mild cognitive impairment; MRI, magnetic resonance imaging; nWBV, normalized whole-brain volume; OS, operating system; PET, positron emission tomography; SaMD, software as a medical device. Created with BioRender.com.

Figure 2 illustrates this translational-readiness framework.

### 2.4 Narrative structure

The synthesis follows the logic of a clinical pathway rather than a technology catalogue.

The review therefore follows a staged logic: screening signal, clinical meaning, biological confirmation, and implementation pathway. A cognitive or digital result alone does not diagnose AD.

## 3 The analog baseline: what brief cognitive screening still does well, and where it fails

Digital cognitive screening should not be presented as a replacement for clinical reasoning, nor as a simple technological upgrade of paper-and-pencil testing. Its value can only be judged against the current diagnostic baseline: brief cognitive tests, informant questionnaires, subjective cognitive complaints, functional assessment, neuropsychiatric symptoms, and the clinical interview. At the same time, they expose the limitations that digital and biomarker-informed approaches are now expected to address.

### 3.1 Brief cognitive tests: useful triage tools, not diagnostic endpoints

For more than four decades, cognitive screening has relied on short instruments designed for rapid administration in primary care, neurology, geriatrics, and memory clinics. The MMSE remains the most historically influential tool, largely because of its simplicity, familiarity, and broad use in clinical care and research (8). Yet its limitations are well established: limited sensitivity to subtle executive, visuospatial, and episodic-memory decline; vulnerability to educational and cultural effects; and ceiling effects in individuals with high premorbid ability or early-stage disease (7, 31).

The MoCA was developed to address some of these shortcomings. Compared with the MMSE, it places greater emphasis on executive function, visuospatial ability, attention, abstraction, and delayed recall (9). This broader cognitive sampling improves sensitivity for MCI, but at the cost of longer administration time, more complex scoring, and possible loss of specificity when cut-offs are not locally calibrated (9, 32).

The Mini-Cog and Clock Drawing Test occupy the opposite end of the spectrum: they are very brief and operationally attractive. The Mini-Cog combines three-word recall with a simplified clock drawing task, thereby adding an episodic-memory component that the clock task alone lacks (10). However, the evidence base remains uneven. A Cochrane review of Mini-Cog in community settings identified only three eligible studies, all conducted by the original developers, with sensitivities ranging from 0.76 to 0.99 and specificities from 0.83 to 0.93; methodological limitations and risk of bias prevented firm recommendations for routine community screening (33).

Spanish evidence provides a useful real-world perspective. In a neurology clinic sample of 581 individuals, Carnero-Pardo et al. (11) reported better diagnostic usefulness for the Mini-Cog than for the Clock Drawing Test, with AUC values of 0.88 and 0.84, respectively. At the 2/3 cut-off, Mini-Cog sensitivity was 0.90 and specificity 0.71, while the Clock Drawing Test at 5/6 showed sensitivity of 0.77 and specificity of 0.80. However, both instruments performed substantially worse in individuals with low educational level, with AUC values around 0.74–0.75. This point is critical for digital translation: digitizing a biased task does not eliminate its bias.

### 3.2 Memory-focused and culturally adapted brief tests: M@T, Fototest, Eurotest, and memory impairment screen (MIS)

Not all brief cognitive tools answer the same clinical question. Some are global multidomain screeners; others deliberately emphasize memory because episodic-memory impairment is a common early feature of typical AD. The M@T was developed as a memory-focused instrument for detecting amnestic MCI and early AD (12). Its strength lies in sensitivity to memory impairment; its limitation is the same. A tool centered mainly on memory may miss non-amnestic presentations, atypical AD phenotypes, vascular cognitive impairment, frontotemporal syndromes, Lewy body disease, or dysexecutive profiles.

The Fototest offers a different model (34). It includes naming, semantic fluency, and free and cued recall, and has been studied in Spanish clinical and primary-care settings as a short, efficient, and culturally pragmatic tool, including in individuals with limited literacy (13). In a phase III primary-care diagnostic accuracy study comparing brief cognitive tests recommended by Spanish clinical guidelines, Eurotest and M@T showed the highest AUCs, 0.91 and 0.90, respectively, but required approximately 7 min. Fototest and Memory Impairment Screen both showed AUCs of 0.87, with Fototest requiring less than half the administration time of Eurotest or M@T (13, 35).

A later population and validation study reinforced this point. In a representative population sample and a validation cohort of individuals consulting for cognitive concerns, Fototest showed the best diagnostic utility among several brief tests, with an AUC of 0.851. Combining Fototest with the informant-based AD8 improved discrimination further, with an AUC of 0.875. The authors proposed more demanding cut-offs for primary-care detection: AD8 ≥ 1, Fototest ≤35, M@T ≤ 40, and MMSE ≤26 (35, 36). These findings are highly relevant for a digital-era review because they show that innovation does not always require more complex technology.

### 3.3 Informant and subjective measures: detecting change, not only impairment

Cognitive screening should not rely only on one-time patient performance. Informant-based tools capture change from a previous level of functioning, which is central to the clinical definition of cognitive decline. The AD8 is a brief informant questionnaire that asks about intra-individual change in memory, judgment, daily functioning, repeated questioning, hobbies, and decision-making (37, 38). Its main value is that it reframes assessment from “How did the patient perform today?” to “Has this person changed?”

This distinction is particularly useful in two common scenarios. Highly educated individuals may perform within the normal range despite early decline because cognitive reserve masks deficits. Conversely, individuals with low educational attainment may score poorly because of lifelong educational disadvantage rather than neurodegeneration. Informant report helps interpret both patterns. The Spanish population study cited above found that AD8 alone performed well and that combining AD8 with Fototest improved diagnostic discrimination (35).

Subjective cognitive complaints provide another entry point. They are often the first reason for consultation and are particularly relevant in remote or digital pathways, where assessment may begin because the person notices change before a clinician does. Subjective cognitive decline is clinically heterogeneous: it may reflect early AD, anxiety, depression, sleep disturbance, medication effects, normal aging, personality traits, or heightened health awareness. Nevertheless, subjective concern should not be dismissed, especially when complaints are persistent, recent, associated with worry, corroborated by an informant, or present in individuals with AD risk factors (39, 40).

Measures such as the Cognitive Concerns Index, Cognitive Complaint Interview, Subjective Cognitive Decline Questionnaire, and related instruments can help operationalize these symptoms (41–43). Their role is not to diagnose MCI or AD, but to identify individuals who may benefit from objective testing, follow-up, informant assessment, or biomarker-based risk stratification. Subjective concern should therefore be interpreted with cognitive performance, informant report, mood, sleep, function, comorbidity, and, when indicated, biomarker evidence.

### 3.4 Functional assessment: the boundary between MCI and dementia

Functional assessment is not optional. It defines the clinical boundary between MCI and dementia and determines the real-world meaning of cognitive impairment. A person may have poor memory performance but preserved independence, supporting a diagnosis of MCI; another may have a similar cognitive profile with loss of instrumental activities of daily living, supporting a diagnosis of dementia. Functional tools are therefore essential in any screening-to-diagnosis pathway.

Commonly used instruments include the Functional Activities Questionnaire, Lawton–Brody Instrumental Activities of Daily Living scale, Barthel Index, Katz Index, Disability Assessment for Dementia, and Interview for Deterioration in Daily Living Activities in Dementia (IDDD) (44–49). IDDD is particularly relevant because it distinguishes initiative from performance in daily activities, helping to clarify whether functional problems arise from cognitive failure, apathy, motor limitation, environmental dependence, or caregiver compensation.

Digital cognitive platforms should therefore incorporate functional assessment rather than treating cognition as an isolated score.

### 3.5 Neuropsychiatric symptoms: the neglected dimension of cognitive screening

Neuropsychiatric symptoms are not secondary details. Apathy, anxiety, depression, irritability, sleep disturbance, agitation, hallucinations, and disinhibition influence cognition, caregiver burden, prognosis, institutionalization, and healthcare use. They may also confound cognitive screening: depression and anxiety can reduce test performance, apathy may mimic executive dysfunction, and sleep disturbance may impair attention and memory.

The Neuropsychiatric Inventory and its brief version, the NPI-Q, remain among the most widely used instruments to quantify behavioral and psychological symptoms in dementia (16, 50). In the Spanish AD burden review, NPI-Q and related neuropsychiatric instruments appeared in studies of mild-to-moderate AD (1, 51).

Digital cognitive tools should not be relied upon to determine the cause of symptoms or to differentiate neurodegeneration from conditions like depression, delirium risk, sleep issues, medication side effects, sensory impairments, or behavioral symptoms independently. Their main role in triage is to measure cognitive performance and provide relevant contextual information—such as mood, sleep quality, neuropsychiatric symptoms, functional status, sensory health, and medication use—so that clinicians can interpret poor performance within the broader clinical picture.

### 3.6 The analog ceiling: why current pathways miss early disease

The main limitation of traditional screening is not just that tests are outdated or paper-based. The core issue is that most were created to identify established cognitive impairment, not biomarker-detectable AD-related change before overt functional decline.

First, ceiling effects happen when high-functioning individuals score within the normal range despite subtle decline. This is especially true for the MMSE but can also affect other brief tests if the task is too easy or the scoring is too narrow. Second, educational and cultural differences distort interpretation: low scores may reflect limited schooling, unfamiliarity with test procedures, or language barriers; high scores may indicate cognitive reserve rather than the absence of disease. Third, testing at a single point in time does not account for intra-individual decline. Someone’s score may be normal compared to population norms but abnormal compared to their own past results. Fourth, manual administration and scoring introduce variability, particularly in drawing tasks, fluency scoring, clock interpretation, timing-dependent tasks, and multilingual environments. Fifth, brief tests seldom combine cognition, function, behavior, subjective complaints, and biomarkers into a single staged process. As a result, screening often remains disconnected from diagnosis, prognosis, and treatment eligibility (7, 31, 39).

### 3.7 What analog tools teach digital screening

Despite their limitations, traditional tools offer four lessons for digital innovation. First, brevity matters: a tool that takes too long will not scale in primary care, regardless of its accuracy. Second, interpretability matters: clinicians understand what recall failure, poor clock drawing, informant-observed decline, functional loss, and neuropsychiatric burden mean. Digital biomarkers must reach a comparable level of clinical intelligibility. Third, context matters: cognitive scores are meaningful only when interpreted alongside education, sensory status, mood, sleep, function, informant report, comorbidity, and medication exposure. Fourth, screening is not diagnosis: a positive screen should trigger a diagnostic pathway; it should not label a person with AD.

This principle is even more important for AI-enabled tools, whose outputs may appear more authoritative than they are. The relevant question is not whether digital tools should replace MMSE, MoCA, Mini-Cog, M@T, Fototest, AD8, IDDD, or NPI. The next stage of cognitive screening should combine the efficiency of brief tests, the longitudinal sensitivity of digital measurement, the ecological meaning of functional assessment, the contextual value of informant report, and the biological specificity of biomarkers.

The instruments discussed in this section are summarized in Table 3.

**Table 3:** Selected non-digital and contextual instruments relevant to early cognitive impairment pathways.

Instrument/family	Primary construct	Typical burden	Most useful role in a staged pathway	Key limitation
MMSE	Global cognition; orientation, recall, attention, language, simple construction	5–10 min	Common reference for global cognitive status, staging, and comparison with historical cohorts	Ceiling effects, limited sensitivity to MCI, and marked educational/cultural dependence
MoCA	Multidomain cognition with stronger executive, attentional, visuospatial, abstraction, and delayed-recall components	10–15 min	First-line screen when MCI is suspected and a more demanding brief test is feasible	Cut-offs require local calibration; higher sensitivity may reduce specificity in low-prevalence settings
ACE-III/ACE-R	Extended bedside multidomain cognitive profile	15–25 min	Intermediate option when more detail than MMSE/MoCA is needed but full neuropsychology is unavailable	Administration time limits scalability in primary care
Mini-Cog	Three-word recall plus clock drawing	<3 min	Very brief triage tool in time-constrained settings	Performance is affected by education and graphomotor demands
Clock Drawing Test	Visuoconstruction, planning, semantic knowledge of clock layout, executive control	1–3 min	Qualitative marker of visuospatial and executive dysfunction	Scoring variability; confounding by education, drawing ability, tremor, and visuomotor impairment
M@T	Episodic and semantic memory; amnestic profiles	6–8 min	Memory-focused screen when typical early AD is suspected	May under-detect non-amnestic, dysexecutive, vascular, Lewy body, or frontotemporal presentations
Fototest	Naming, semantic fluency, free recall, and cued recall	~3 min	Efficient brief screen in Spanish-speaking and lower-literacy contexts	Requires local norms and cut-off calibration; not etiologically specific
Eurotest	Currency handling, calculation, memory, and executive components	~7 min	Practical cognitive screen incorporating everyday numerical and executive demands	Performance may be influenced by numerical confidence and familiarity with currency tasks
MIS	Controlled encoding, free recall, and cued recall	~4 min	Short memory screen with controlled learning conditions	Limited coverage outside memory; not suitable for all low-literacy individuals
AD8/IQCODE-type tools	Informant-observed intra-individual cognitive or functional change	2–5 min	Contextualizes current performance against previous functioning and cognitive reserve	Requires a reliable informant; responses may reflect caregiver burden or limited observation
Subjective complaint scales	Patient-perceived cognitive change	Variable	Identifies help-seeking individuals and possible pre-MCI risk states	Non-specific; influenced by mood, anxiety, sleep, personality, and health beliefs
FAQ/IDDD/Lawton–Brody IADL	Instrumental functional change linked to cognition	5–15 min	Distinguishes MCI from dementia and links cognition to everyday independence	Informant-dependent; affected by frailty, physical disability, apathy, depression, and environment
Barthel/Katz Index	Basic activities of daily living and dependency	5–10 min	Quantifies dependency and care burden, especially in established dementia	Less sensitive to subtle early instrumental decline
NPI/NPI-Q	Neuropsychiatric and behavioral symptoms	5–15 min	Identifies behavioral burden and treatable confounders affecting cognition and care needs	Not specific to AD; symptoms may mimic, precede, or obscure cognitive decline
CDR/GDS	Global clinical severity and functional staging	5–20 min	Supports staging and longitudinal clinical classification	Staging tools are not etiological tests and may be insensitive to subtle prodromal change

Tools differ in construct, burden, and clinical role and should be interpreted as complementary rather than interchangeable. ACE, Addenbrooke’s Cognitive Examination; AD, Alzheimer’s disease; AD8, Ascertain Dementia 8; CDR, Clinical Dementia Rating; FAQ, Functional Activities Questionnaire; GDS, Global Deterioration Scale; IADL, instrumental activities of daily living; IDDD, Interview for Deterioration in Daily Living Activities in Dementia; IQCODE, Informant Questionnaire on Cognitive Decline in the Elderly; MCI, mild cognitive impairment; MIS, Memory Impairment Screen; MMSE, Mini-Mental State Examination; MoCA, Montreal Cognitive Assessment; NPI, Neuropsychiatric Inventory; M@T, Memory Alteration Test.

## 4 Digital cognitive assessment: from digitized tests to digital cognitive biomarkers

The digital transformation of cognitive screening has followed two partly overlapping trajectories. The first is the digitization of established instruments, in which conventional tests are transferred to tablets, computers, web platforms, or mobile devices while preserving much of the original task structure. The second is the development of native digital assessments, which exploit signals that are largely inaccessible to analog testing: millisecond-level response timing, graphomotor dynamics, eye movements, speech acoustics, natural-language features, repeated high-frequency sampling, remote administration, and machine-learning–based classification (19, 21, 52).

This distinction is essential. A digital MMSE, digital MoCA, or tablet-based clock drawing test may improve standardization and reduce scoring error, but it does not automatically solve the psychometric limitations of the original instrument. Conversely, native digital tools may capture more sensitive features of cognitive performance, but often require more extensive validation before they can be trusted in clinical pathways (21, 53).

**Figure 3:**
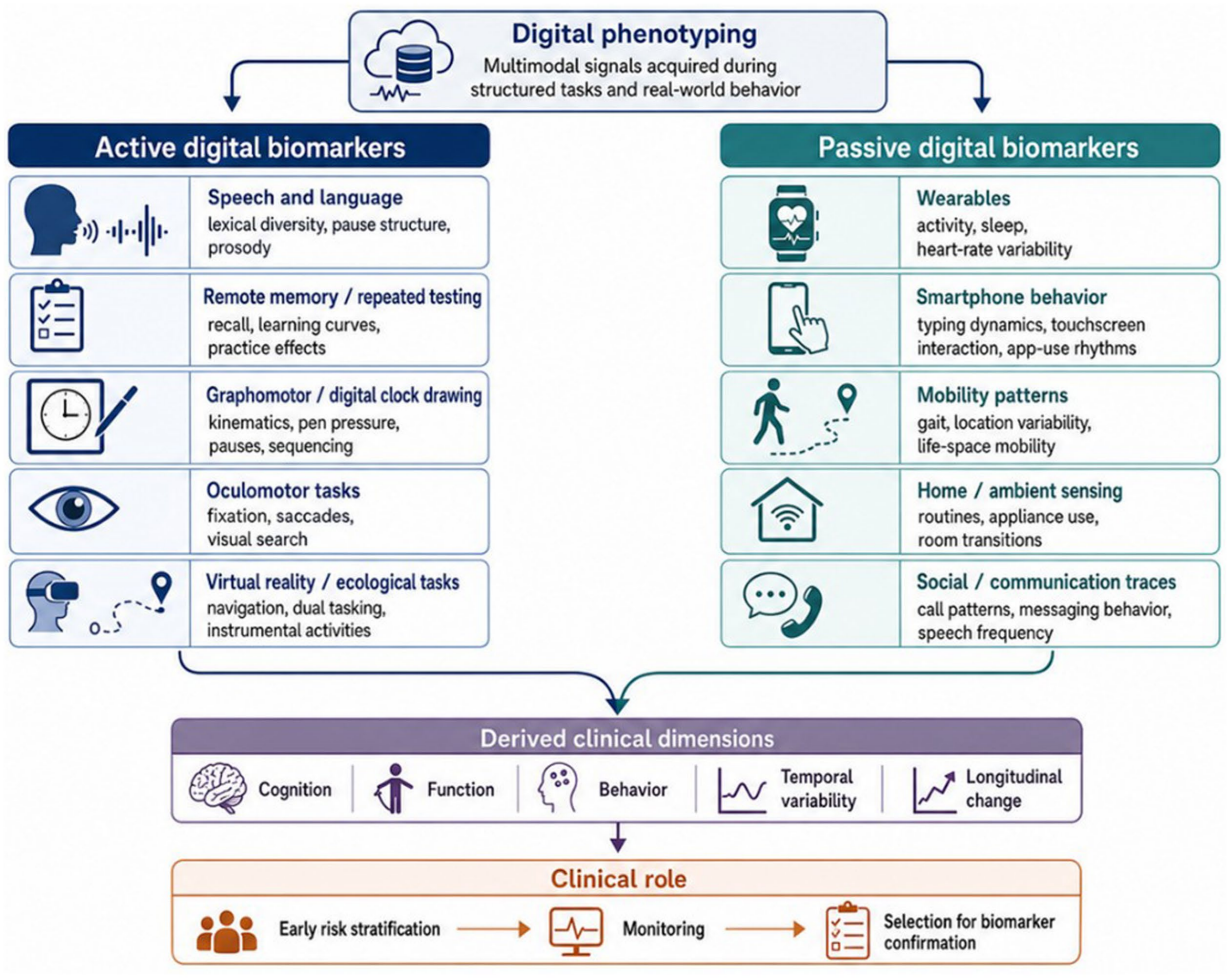
Active and passive digital biomarkers for early cognitive phenotyping. Active biomarkers arise from structured digital tasks; passive biomarkers are inferred from routine device, mobility, wearable, or ambient signals. Both may support risk stratification and longitudinal monitoring, but require clinical contextualization before confirmatory biomarker referral. Created with BioRender.com. VR, virtual reality.

A second distinction is equally important: active digital assessment versus passive or semi-passive digital phenotyping. Active assessments are bounded tasks that the individual knowingly performs, such as remote memory testing, speech sampling, digital drawing, reaction-time tasks, virtual navigation, or prospective-memory paradigms. Passive or semi-passive approaches infer cognitive risk from everyday behavior or sensor-derived signals, including keystrokes, device use, gait, mobility, sleep, circadian rhythm, GPS patterns, wearable data, or ambient sensing (21, 54, 55). Figure 3 summarizes this distinction between active digital tasks and passive or semi-passive digital phenotyping, emphasizing that both types of signal require clinical contextualization before they are used for risk stratification, monitoring, or biomarker referral.

### 4.1 Digitized conventional tests and computerized batteries

Digitized versions of traditional instruments offer immediate operational advantages. Automated instructions reduce examiner variability; digital scoring can minimize arithmetic and interpretation errors; embedded timers capture response latency; and remote platforms can support decentralized assessment.

Examples include computerized or tablet-based versions of the MoCA, MMSE, Clock Drawing Test, Trail Making paradigms, Cogstate, CANTAB, NIH Toolbox Cognition Battery, TabCAT Brain Health Assessment, BrainCheck, and related platforms (52, 56–60).

Digital clock drawing illustrates the added value of process data. Analog scoring is usually based on the final drawing. Digital versions can quantify how the drawing was produced: stroke order, latency before drawing, pauses, pen lifts, velocity, pressure, spatial organization, sequencing, and self-corrections. These hidden metrics may reveal executive, visuospatial, or graphomotor inefficiency before the final clock appears frankly abnormal. Studies of digital clock drawing have also linked graphomotor features with amyloid and tau PET measures in cognitively normal older adults, suggesting that fine-grained motor-executive behavior may carry information beyond conventional scoring (22, 61).

Computerized batteries provide another model. Their strengths include standardized administration, domain-specific profiling, and repeatability. In cognitively normal older adults with known amyloid PET status, remote and in-clinic digital measures have shown promising discrimination of amyloid positivity. For example, smartphone-based associative-memory tasks and TabCAT. Favorites have performed at least comparably to MoCA in selected biomarker-enriched samples (23, 56). These findings do not make such tools diagnostic of AD pathology, but they support their possible role in early-risk enrichment when embedded in an appropriate confirmatory pathway.

Digitization should therefore not be equated with transformation. If a task is biased by education, language, motor impairment, visual impairment, test familiarity, or symbolic conventions, a digital version may preserve the same bias and add device-related confounding. Digital tools must therefore be validated not only against diagnostic labels, but also across the populations and settings in which they are intended to be used.

### 4.2 Remote repeated assessment and measurement burst paradigm designs

One of the most important innovations in digital cognitive assessment is the move from single-session testing to repeated measurement. This is not merely a logistical advantage. Cognitive performance varies with sleep, mood, fatigue, stress, medication, pain, time of day, and environmental context. A single clinic score may provide a noisy estimate of cognitive capacity. Repeated digital assessment can estimate mean performance, intra-individual variability, short-term learning, practice effects, fatigue, and temporal fluctuation (21, 62). Repeated assessment and measurement-burst designs should nevertheless be distinguished. Repeated assessment refers broadly to cognitive testing repeated over time, for example, at monthly, quarterly, or six-month intervals, and is primarily suited to estimating within-person change. A measurement-burst design refers to multiple brief assessments collected within a short window, which can improve reliability and quantify intra-individual variability; a burst may be used cross-sectionally or repeated longitudinally. Both designs are relevant to digital cognitive assessment, but they answer different questions: repeated assessment estimates change, whereas burst designs estimate short-term variability, context dependence, and aggregate performance.

The Mobile Monitoring of Cognitive Change platform (M2C2) illustrates a measurement-burst approach. In a fully remote protocol, cognitively healthy older adults completed brief app-based cognitive sessions multiple times per day over several days. Adherence was high in selected participants, and aggregated within-person reliabilities were strong across episodic memory, working memory, and processing-speed tasks (63, 64). This approach has been extended to biomarker-enriched samples. Among cognitively normal older adults with known amyloid PET status, memory-focused digital tasks such as M2C2 Prices and TabCAT. Favorites showed promising ability to distinguish amyloid-positive from amyloid-negative individuals (23). The value of these tasks lies not only in baseline accuracy but in their ability to capture learning and practice-related change. In normal cognition, repeated exposure often improves performance. Failure to benefit from practice may indicate early cognitive vulnerability, particularly in preclinical or prodromal AD (62, 65).

Both repeated and burst-based digital protocols introduce practical and psychometric challenges. High adherence in research cohorts may not generalize to older adults with low digital literacy, sensory impairment, depression, low motivation, limited internet access, or unstable home environments. These designs require alternate forms, careful handling of practice effects, and statistical models that distinguish true cognitive change from fatigue, distraction, stimulus familiarity, or device problems.

### 4.3 Speech, language, and conversational AI

Speech and language are among the most promising modalities for deriving digital cognitive markers, because connected speech integrates several cognitive and neural systems. In this context, the candidate digital markers are not “speech” itself, but quantifiable acoustic, temporal, lexical, semantic, syntactic, and discourse-level features derived from speech samples. Producing connected speech requires semantic memory, lexical retrieval, working memory, executive control, articulation, respiratory coordination, prosody, and discourse planning. Cognitive decline may therefore affect both what is said and how it is said (20, 66–69).

Linguistic markers include reduced lexical diversity, lower semantic specificity, increased use of pronouns or fillers, simplified syntax, repetitions, word-finding difficulty, and reduced discourse coherence. Acoustic and temporal markers include slower speech rate, longer pauses, altered pause distribution, articulation changes, jitter, shimmer, and prosodic flattening. These features can be extracted from picture description, story recall, semantic fluency, autobiographical narrative, procedural discourse, or spontaneous conversation (66, 68, 69).

The study by Hilsabeck et al. (20) is particularly relevant because it combined a short working-memory/processing-speed task with connected speech analysis for possible primary-care use. In 53 cognitively normal and 51 cognitively impaired older adults, the best-performing model combined two administrations of a speeded matching task with acoustic and linguistic features from a personal narrative task, achieving an AUC of 0.84, slightly above the Quick Mild Cognitive Impairment screen (20). The most informative variables included initial speeded matching performance, practice-related change, type-token ratio, and acoustic features related to voice quality.

Voice-based digital cognitive screeners may also reduce barriers in community settings. The Chinese Digital Cognitive Screener evaluates delayed word recall, orientation, and semantic fluency using a voice-recognition interface. Reported validation data show short administration time, high completion rates, and strong discrimination for dementia and MCI in community-dwelling older adults (70, 71). A technically important aspect of this work is the transition from a two-step automatic speech recognition plus natural-language-understanding pipeline to multimodal approaches that align audio features more directly with expected response content.

The practical appeal is clear. Many older adults are more comfortable speaking than using a touchscreen, keyboard, or mouse. Voice-based tools may therefore reduce part of the digital-literacy burden and could be administered with minimal support by non-specialist facilitators. Yet speech tools require particularly careful local validation. Language, dialect, accent, hearing loss, speech disorder, depression, education, cultural narrative style, microphone quality, and background noise can all influence performance.

### 4.4 Prospective memory and ecological digital tasks

Most brief screening tools emphasize orientation, recall, clock drawing, or verbal fluency. However, many everyday cognitive failures involve prospective memory: remembering to perform an intended action at the appropriate time or in response to a cue. This ability is directly relevant to medication adherence, appointments, cooking safety, financial tasks, and independent living (72, 73).

The Chrono pilot study addresses this underexplored domain. Chrono is a digital tool based on event-based prospective memory, using focal and non-focal cues embedded within ongoing tasks. In a validation sample of 67 individuals classified according to MoCA cut-off as probably impaired or not impaired, the Chrono score achieved an AUC of 0.813. At the optimal cut-off of 2.5, sensitivity was 0.83 and specificity was 0.64. Chrono performance correlated with neuropsychological measures and executive components such as updating and shifting (74).

Digital platforms are particularly suited to prospective-memory assessment because they can embed cues, distractions, timing constraints, and competing tasks within interactive scenarios. Unlike a simple word-recall task, a prospective-memory task can approximate real-world situations in which the person must remember an intention while engaged in another activity. The challenge is usability. Instructions may be complex, older adults with low digital familiarity may be disadvantaged, and visual or motor limitations may affect performance. Ecological relevance must therefore be balanced against accessibility.

### 4.5 Virtual reality, serious games, and simulated everyday tasks

Virtual reality and serious games extend ecological assessment further. Rather than asking isolated questions, they place the participant in a simulated environment requiring integrated cognition: remembering goals, navigating space, inhibiting distractions, planning sequences, switching attention, and completing everyday tasks. Examples include the Virtual Supermarket, Panoramix, virtual maze tasks, digital kiosk tasks, navigation games, and other gamified cognitive platforms (19, 75–78).

The rationale is compelling. Early cognitive decline often appears first in complex instrumental activities rather than in simple orientation questions. Serious games may increase engagement and reduce the artificiality of clinic testing. Recent studies and reviews suggests that several virtual-reality and serious-game tools show promising diagnosis signals for MCI detection, but the evidence remains heterogeneous in sample size, reference standards, cognitive domains, and validation depth (19, 78).

The limitation is equally clear. High ecological validity does not guarantee diagnostic validity. Many virtual-reality and serious-game studies remain small, exploratory, and conducted in selected samples. Hardware, screen size, headset tolerability, motion sickness, visual impairment, manual dexterity, and prior gaming experience may all affect performance. A simulated shopping task may be clinically intuitive, but it must still demonstrate reliability, test–retest stability, normative calibration, cross-cultural validity, and incremental value beyond conventional cognitive and functional measures.

### 4.6 Eye tracking, graphomotor metrics, keystroke dynamics, and passive sensing

A growing body of work has explored digital markers that are not traditional cognitive scores. Eye tracking can quantify fixation duration, saccadic latency, gaze transitions, visual exploration, attentional disengagement, and visual search strategies. Graphomotor analysis captures drawing speed, pressure, pauses, trajectory, stroke order, and tremor-like features. Keystroke dynamics can assess typing latency, error correction, inter-key intervals, and motor-cognitive rhythm. Wearables and ambient technologies can provide information on sleep, circadian activity, gait, heart rate variability, physical activity, mobility range, and daily routines (21, 54, 55, 79–81).

These signals may capture subtle neurocognitive change in real-world conditions, but their interpretation is vulnerable to non-cognitive confounding. Eye tracking may be affected by ocular disease, glasses, lighting, screen calibration, and fatigue. Graphomotor features may reflect arthritis, tremor, neuropathy, handedness, vision, or stylus familiarity. Keystroke, wearable, and mobility-derived metrics may reflect frailty, depression, sleep apnea, pain, medication, comorbidity, device habits, or lifestyle rather than neurodegeneration.

Passive or semi-passive phenotyping also raises ethical concerns because cognitive risk may be inferred from routine behavior outside a bounded clinical task. Key issues include consent granularity, data minimization, identifiability of raw behavioral traces, secondary use, household privacy, commercial access, and cognitive surveillance.

### 4.7 Validation beyond accuracy

Digital cognitive tools require staged, use-dependent validation. A high AUC in a selected sample is not enough. The evidentiary standard should reflect the intended use of the tool: population screening, primary-care triage, remote monitoring, trial enrichment, biomarker referral, or treatment-readiness support. Across these uses, several domains are usually relevant: technical validity, psychometric stability, construct validity, diagnostic accuracy, biomarker or etiological validity, longitudinal sensitivity, clinical actionability, equity, interpretability, and implementation feasibility.

Evidence in the field is progressing beyond feasibility. Digital clock drawing has been associated with amyloid and tau PET, remote and unsupervised memory assessments have shown associations with AD biomarkers and cognitive decline, and high-frequency app-based protocols have demonstrated encouraging reliability and adherence in selected cohorts (22, 23, 82–85) Early implementation studies are also beginning to examine how digital tools perform in real clinical pathways rather than only in research cohorts (86). These examples are encouraging, but they do not remove the need for external validation, subgroup calibration, longitudinal follow-up, and predefined clinical action rules. A tool designed to monitor change in subjective cognitive decline does not require the same evidentiary package as a tool intended to prioritize biomarker referral or support treatment-readiness assessment.

### 4.8 Clinical synthesis

Digital cognitive assessment is best understood as an expansion of cognitive phenotyping rather than a single replacement test. Its promise lies in four areas: standardization, granularity, scalability, and longitudinal measurement. Its risks include overstating performance from selected samples, poor generalization to new settings, systematic errors in underrepresented groups, exclusion of people with limited digital access or sensory impairment, misuse of sensitive cognitive or behavioral data, and premature reliance on automated outputs without adequate clinical oversight (19, 21, 87).

The most advanced digital tools are characterized by easy-to-use interfaces, clear theoretical models, reliable psychometric properties, and validation through clinical or biomarker outcomes. Conversely, less developed tools rely on small sample sizes, opaque algorithms, device-specific metrics, and lack external or longitudinal validation.

Table 4 summarizes the main digital cognitive assessment modalities reviewed in this section, emphasizing the type of signal captured, the evidence base currently supporting each approach, and the limitations that should be addressed before routine clinical use.

**Table 4:** Main digital cognitive assessment modalities reviewed according to signal type, current evidence, plausible near-term clinical role, and unresolved translational barriers.

Modality	Representative examples	Primary signal	Current evidence base	Most defensible near-term role	Key unresolved issue
Digitized conventional tests	Electronic MMSE/MoCA, tablet MMSE, digital Clock Drawing Test, digital Trail Making paradigms	Conventional task performance plus timing, error, sequence, and response-process metrics	Feasibility and convergent validity are generally available; strongest when digital delivery reduces scoring variability or captures process data	Standardized administration, semi-remote screening, automated scoring, and reduction of examiner-dependent error	May preserve the educational, cultural, sensory, or motor biases of the original task
Computerized cognitive batteries	Cogstate, CANTAB, NIH Toolbox, TabCAT, BrainCheck	Domain-level memory, attention, processing speed, executive function, visuospatial ability, associative learning	More mature psychometric base than many newer digital tools; some clinical and biomarker associations	Research assessment, trial enrichment, memory-clinic support, and structured cognitive profiling	Normative calibration, licensing, infrastructure, and interpretability in non-specialist settings
Remote repeated assessment	M2C2, remote memory composites, neotiv-like paradigms, ARC/BRANCH-type approaches	Mean performance, intra-individual variability, practice effects, learning curves, time-of-day effects	Feasibility and reliability are encouraging in selected cohorts; biomarker-linked evidence is emerging	Longitudinal monitoring, early-risk enrichment, and detection of subtle change not visible in single-session testing	Adherence bias, environmental variability, practice-effect modelling, and limited validation in digitally less confident populations
Speech, language, and conversational AI	Connected-speech classifiers, voice-based cognitive screeners, DCS-like systems, IA Voce-type platforms	Acoustic, temporal, lexical, semantic, syntactic, and discourse-level markers	Rapidly expanding cross-sectional evidence; some tools show promising discrimination of MCI or dementia	Low-burden triage, remote screening, and adjunctive phenotyping in primary care or community pathways	Language, dialect, accent, hearing loss, microphone quality, depression, and model opacity require local validation
Digital graphomotor analysis	DCT clock, digital figure copy, digital Trail Making, stylus-based drawing tasks	Drawing latency, stroke order, pauses, velocity, pressure, spatial organization, correction behavior	Promising process-level signal, especially when final paper score is normal but execution is inefficient	Adjunctive detection of subtle executive, visuospatial, and motor-planning changes	Confounding by tremor, arthritis, visual impairment, handedness, stylus familiarity, and device differences
Oculomotor and gaze-based measures	Eye tracking during memory, attention, visual exploration, or virtual tasks	Fixation duration, saccade latency, gaze transitions, attentional disengagement, visual search strategy	Biologically plausible and technically rich, but validation remains heterogeneous	Research phenotyping and adjunctive assessment in controlled settings	Calibration burden, ocular disease, lighting, glasses, fatigue, and lack of widely accepted clinical thresholds
Ecological active tasks	Virtual Supermarket, Panoramix, virtual maze/navigation tasks, serious games, Chrono/prospective-memory paradigms	Multitask performance, spatial navigation, intention maintenance, planning, divided attention, cue detection	Pilot and proof-of-concept studies often show promising diagnostic signals but small and heterogeneous samples	Ecological assessment, functional-risk enrichment, and research on everyday cognition	Hardware burden, instruction complexity, motion sickness, prior gaming experience, and uncertain generalizability
Wearable and passive or semi-passive sensing	Keystroke dynamics, gait/mobility, sleep/circadian rhythm, GPS patterns, wearable activity, smart-device behavior	Real-world behavioral rhythms, motor-cognitive efficiency, activity regularity, sleep architecture, mobility change	Mostly exploratory for cognitive screening; feasibility evidence is stronger than diagnostic actionability	Longitudinal monitoring and hypothesis generation, particularly when combined with active cognitive or clinical data	Consent, privacy, surveillance risk, lifestyle confounding, multimorbidity, missing data, and unclear clinical thresholds
Multimodal digital classifiers	Cognitive task + speech models, digital phenotype + informant data, AI models integrating behavioral and biomarker signals	Integrated risk profile across cognition, language, motor behavior, function, and sometimes biological data	Conceptually strong and increasingly reported, but vulnerable to overfitting and limited external replication	Risk stratification and prioritization for biomarker confirmation or specialist assessment	External validation, calibration, explainability, fairness testing, and predefined clinical-action rules

Evidence maturity should not be inferred from technological complexity alone. AI, artificial intelligence; CANTAB, Cambridge Neuropsychological Test Automated Battery; DCS, Digital Cognitive Screener; DCTclock, digital Clock Drawing Test; M2C2, Mobile Monitoring of Cognitive Change; MCI, mild cognitive impairment; MMSE, Mini-Mental State Examination; MoCA, Montreal Cognitive Assessment.

Taken together, these modalities show that digital assessment is not a diagnostic shortcut. Its strongest contribution lies in combining standardization, process-level measurement, ecological sampling, and longitudinal follow-up.

## 5 Biological anchoring and the contested diagnostic boundary in the therapeutic era

Digital cognitive screening becomes clinically meaningful only when it is connected to a diagnostic pathway. A cognitive score, speech-derived risk estimate, graphomotor feature, eye-tracking marker, or app-based memory trajectory may change the probability of cognitive impairment, but none of these signals can establish AD etiology by itself. This distinction is central in the current therapeutic era. Anti-amyloid monoclonal antibodies have increased the clinical value of early detection, but they have also raised the evidentiary threshold for diagnosis, staging, safety assessment, and treatment eligibility (5, 6).

The relevance of biological anchoring to digital screening lies in the escalation decision. Digital measures may identify individuals whose cognitive, linguistic, graphomotor, functional, or longitudinal profile warrants further evaluation, but their clinical value depends on whether they improve the selection of patients for biomarker testing, specialist assessment, longitudinal monitoring, or treatment-readiness evaluation. Digital screening therefore sits between a pragmatic need for scalable early case-finding and an ethical obligation to avoid etiological labeling, risk disclosure, or high-burden diagnostic procedures without adequate clinical and biological grounding.

### 5.1 Why cognitive screening requires biological anchoring

Traditional and digital cognitive tests measure performance, not pathology. A low cognitive score may reflect AD, vascular cognitive impairment, Lewy body disease, frontotemporal degeneration, depression, anxiety, poor sleep, delirium, medication effects, sensory impairment, low literacy, or unfamiliarity with the testing procedure. Conversely, a normal score may occur in individuals with high cognitive reserve who already show amyloid, tau, or neurodegenerative changes (24, 25, 88, 89). Biological anchoring is therefore necessary when screening results are used to guide biomarker referral, specialist assessment, or treatment-readiness decisions. Without this step, digital tools may scale syndromic uncertainty rather than resolve it.

### 5.2 Blood-based biomarkers as a second-line triage layer

Blood-based biomarkers are changing the diagnostic pathway because they offer a less invasive and more scalable bridge between cognitive screening and specialist confirmation. Plasma phosphorylated tau markers, particularly p-tau217 and p-tau181, amyloid beta ratios, glial fibrillary acidic protein, and neurofilament light are increasingly used to estimate AD-related pathology, neurodegeneration, glial activation, or axonal injury (36, 90–93). Their most defensible immediate role is not indiscriminate population screening of asymptomatic individuals, but triage and diagnostic support in people with cognitive symptoms or abnormal screening findings.

The 2025 Alzheimer’s Association clinical practice guideline on blood-based biomarkers is directly relevant to digital screening pathways. It proposes that blood-based biomarker tests with at least 90% sensitivity and 75% specificity can be used as triage tests, while tests reaching at least 90% sensitivity and specificity may substitute for amyloid PET or CSF AD biomarker testing in selected patients with cognitive impairment evaluated in specialized memory-care settings (91, 92). The guideline also cautions that test performance varies and that blood-based biomarkers should not replace comprehensive clinical evaluation (88, 94).

This tiered model fits naturally with digital screening. A brief or digital cognitive screen may identify individuals who should undergo plasma biomarker testing. Plasma biomarker results may then help determine who requires MRI, CSF, amyloid PET, tau PET, or specialist review. Yet blood biomarkers are not a diagnostic shortcut. Their predictive value depends on the assay, cut-off, pretest probability, age, renal function, comorbidity, clinical setting, and whether the population being tested resembles the validation cohort. In low-prevalence settings, false positives and uncertain positives remain clinically important. Digital and plasma results should therefore be interpreted together with phenotype, informant report, function, neuropsychiatric context, and longitudinal course.

### 5.3 Structural MRI and normalized whole-brain volume: useful but non-specific

Structural MRI remains central to the workup of cognitive impairment. It helps exclude alternative or contributing causes, estimates vascular burden, identifies regional atrophy patterns, and informs safety decisions in treatment pathways. Medial temporal atrophy and hippocampal volume are particularly relevant in typical amnestic AD, whereas frontal, language, posterior cortical, vascular, or mixed patterns may suggest alternative etiologies or comorbid disease (24, 95, 96).

MRI-derived markers such as normalized whole-brain volume (nWBV) can quantify global atrophy after accounting for intracranial volume. This is relevant to the ceiling-effect problem. Individuals with normal or near-normal MMSE scores may still show structural vulnerability (97). A high cognitive score may reflect preserved function or cognitive reserve, but it does not guarantee structural integrity. In this context, nWBV and related MRI measures may help identify latent vulnerability: biological degeneration may already be present despite apparently preserved brief-test performance (98–100).

However, structural MRI is not etiologically specific for AD. Global atrophy may reflect age-related change, vascular disease, mixed pathology, traumatic brain injury, alcohol-related injury, inflammatory disease, or other neurodegenerative processes. nWBV should therefore not be treated as an AD diagnostic marker in isolation.

### 5.4 Diagnostic criteria: biological definition versus clinical-biological construct

The 2024 Alzheimer’s Association revised criteria define AD biologically rather than by clinical syndrome alone. In this framework, abnormality on an early-changing “Core 1” biomarker is considered sufficient to establish a diagnosis of AD and to inform clinical decision-making across the disease continuum; the revised framework also incorporates accurate plasma biomarkers into the biomarker classification system (24).

The International Working Group, led by Dubois and colleagues, takes a more conservative clinical-biological position. It argues that, in routine clinical practice, AD should be diagnosed when a compatible clinical phenotype is supported by biomarkers (25). Biomarker-positive cognitively unimpaired individuals should generally be considered at risk, rather than diagnosed with AD solely on the basis of biological abnormality.

This disagreement is not semantic. It determines how digital tools should be deployed. Under a primarily biological framework, digital tools may contribute to staging, monitoring, or functional phenotyping after biomarker abnormality is established. Under a clinical-biological framework, digital tools have a stronger upstream role: identifying meaningful cognitive or functional change that justifies biomarker testing.

For this review, the most defensible position is that digital cognitive screening should be syndrome-sensitive but etiology-aware.

### 5.5 Disease-modifying therapy makes early detection more valuable and more difficult

The therapeutic context has changed rapidly. In the United States, lecanemab received traditional FDA approval in 2023 for AD, with initiation recommended in patients with MCI or mild dementia stage disease, the population studied in trials; treatment requires confirmation of amyloid beta pathology before initiation. Donanemab was approved by the FDA in 2024 for AD, likewise with initiation recommended in patients with MCI or mild dementia stage disease (101, 102).

In Europe, the regulatory position is more restrictive. EMA information for lecanemab describes use in adults with MCI or mild dementia due to AD who have amyloid beta plaques and who have one or no copy of APOE ε4, reflecting the central role of amyloid confirmation and ARIA-risk stratification in the benefit–risk assessment. EMA information for donanemab similarly describes use in adults with MCI or mild dementia due to AD who are APOE ε4 heterozygotes or non-carriers with confirmed amyloid pathology (103).

This creates a practical paradox. Health systems need earlier detection to identify people who may benefit from treatment assessment. Yet earlier detection also increases demand for plasma biomarkers, MRI safety screening, specialist review, infusion infrastructure, ARIA monitoring, disclosure counseling, and reimbursement decisions.

The therapeutic implications of early detection make it necessary to distinguish screening signals from biological confirmation and treatment-readiness markers. Table 5 summarizes the main biological anchors and treatment-relevant decision points that may follow a positive digital or brief cognitive screen.

**Table 5:** Biological and treatment-relevant layers that may follow an abnormal brief or digital cognitive screen.

Layer	Examples	Clinical contribution	Key limitation	Role after positive screening
Digital cognitive phenotype	Speech/language markers, remote memory tasks, digital clock drawing, reaction-time measures, eye tracking, VR tasks, wearables	Identifies scalable behavioral signals of cognitive vulnerability, especially when repeated or combined with informant and functional data	Not etiologically specific; cannot diagnose AD independently	Triage, longitudinal monitoring, and prioritization for clinical or biomarker assessment
Plasma biomarker triage	p-tau217, p-tau181, Aβ42/40, GFAP, NfL	Provides a minimally invasive estimate of AD-related pathology, neurodegeneration, or glial/axonal injury	Assay performance, thresholds, predictive value, and interpretation vary by platform and setting	Second-line triage before CSF/PET or specialist referral in symptomatic or screen-positive individuals
Structural MRI	Hippocampal volume, medial temporal atrophy, cortical atrophy, vascular burden, microbleeds, superficial siderosis, nWBV	Assesses neurodegeneration, vascular contribution, alternative diagnoses, brain reserve, and treatment safety	Atrophy and vascular changes are not specific to AD	Differential diagnosis, risk contextualization, and safety screening before anti-amyloid therapy pathways
CSF biomarkers	Aβ42/40, p-tau, total tau	Provides established biological evidence of amyloid and tau pathology	Invasive; requires expertise, local cut-offs, and careful counseling	Etiological confirmation when clinical syndrome and non-invasive findings are suggestive or discordant
Amyloid PET	Amyloid plaque imaging	Confirms or excludes cerebral amyloid deposition	Cost, access, radiation exposure; positivity does not prove symptom causality	Resolution of diagnostic uncertainty and support for treatment-eligibility assessment
Tau PET	Regional tau imaging	Refines topographic staging and prognostic interpretation	Limited availability, cost, and evolving thresholds	Specialist-level staging, atypical presentations, or research use
APOE genotyping	APOE ε4 status	Refines ARIA-risk stratification and counseling in anti-amyloid treatment pathways	Not diagnostic of AD; genetic counseling and family implications required	Treatment-risk assessment when results alter eligibility, counseling, or monitoring
Treatment-safety monitoring	Baseline/follow-up MRI, ARIA surveillance, vascular/hemorrhagic exclusion criteria	Determines whether disease-modifying therapy can be considered and monitored safely	Safety eligibility does not guarantee individual benefit	Converts etiological diagnosis into treatment-readiness assessment and shared decision-making

Digital phenotypes support triage but do not establish AD etiology; biological and structural markers should be interpreted within the clinical syndrome. AD, Alzheimer’s disease; APOE, apolipoprotein E; ARIA, amyloid-related imaging abnormalities; Aβ, amyloid beta; CSF, cerebrospinal fluid; GFAP, glial fibrillary acidic protein; MRI, magnetic resonance imaging; nWBV, normalized whole-brain volume; NfL, neurofilament light; PET, positron emission tomography; p-tau, phosphorylated tau; VR, virtual reality.

### 5.6 Overdiagnosis, uncertainty, and staged disclosure

Earlier detection has clear potential benefits: timely explanation, identification of reversible contributors, trial referral, caregiver preparation, advance care planning, vascular and lifestyle risk management, and assessment for treatment eligibility (24, 25, 104, 105) It also carries risks: false positives, anxiety, stigma, insurability concerns, unnecessary biomarker testing, and medicalization of individuals whose prognosis remains uncertain (88, 106, 107).

These risks are particularly important when digital and biomarker information are combined. A digital score may suggest elevated risk, a plasma biomarker may suggest AD pathology, and an MRI may show atrophy; however, the clinical meaning of these findings depends on symptoms, function, age, comorbidity, patient preferences, and diagnostic certainty. Disclosure should therefore be staged: Patients should first understand that a screening result is not a diagnosis, and confirmatory testing should be accompanied by counseling on the distinction between risk, pathology, syndrome, prognosis, and treatment eligibility (107–109).

The right not to know should also be respected. Digital platforms should not return uncontextualized cognitive-risk or biomarker-risk scores directly to patients without a clear clinical disclosure pathway and human oversight (107, 109).

### 5.7 From digital signal to biological confirmation: a staged pathway

A responsible pathway begins with low-burden detection and narrows progressively toward higher-specificity, higher-consequence assessments. Initial concern may arise from the patient, an informant, or primary care observation. Brief cognitive and contextual assessment should then establish whether there is objective impairment, informant-confirmed change, functional impact, neuropsychiatric burden, or potentially reversible contributors. Digital phenotyping may add repeated, process-level, or ecologically sampled signals that refine risk stratification.

Biological triage with plasma biomarkers and MRI can help determine whether specialist referral, CSF, amyloid PET, tau PET, or advanced imaging is warranted. Specialist confirmation should integrate clinical syndrome, neuropsychology, imaging, CSF or PET biomarkers, comorbidity, vascular burden, and patient values (24, 88) The final stage is clinical action: reassurance and monitoring, etiological diagnosis, staging, shared decision-making, treatment-readiness assessment, caregiver support, or referral to research (103, 104).

**Figure 4:**
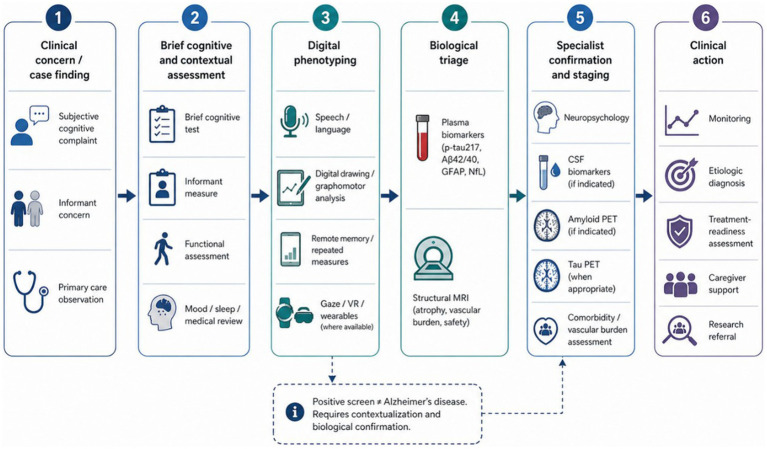
From digital cognitive screening to biological confirmation and clinical action. Stepwise pathway linking clinical concern, brief contextual assessment, digital phenotyping, biological triage, specialist confirmation, and clinical action. A positive digital or brief cognitive screen should not be interpreted as an etiological diagnosis; it requires clinical contextualization and, when indicated, biomarker confirmation before Alzheimer’s disease labeling or treatment-readiness decisions. Created with BioRender.com. Aβ, amyloid beta; CSF, cerebrospinal fluid; GFAP, glial fibrillary acidic protein; MRI, magnetic resonance imaging; NfL, neurofilament light; PET, positron emission tomography; p-tau, phosphorylated tau; VR, virtual reality.

Figure 4 summarizes this proposed escalation pathway.

## 6 Implementation, equity, clinical trust, and governance

A digital cognitive screening tool should not be considered clinically useful simply because it reports a high area under the receiver operating characteristic curve. Clinical value depends on whether the tool can be completed by the intended users, interpreted by clinicians, embedded in a realistic care pathway, and governed safely. In early cognitive impairment, this requirement is especially important because screening results may influence whether a person is reassured, monitored, referred, tested for biomarkers, labeled as being at risk, or considered for disease-modifying therapy.

Implementation validity should therefore be treated as part of the evidence base, not as a late administrative step after psychometric validation. A tool may discriminate well in a selected research sample and still fail in primary care if it generates unmanageable false-positive referrals, excludes people with low digital access, produces outputs that clinicians do not trust, or triggers anxiety without a confirmatory pathway (110, 111).

### 6.1 Implementation validity: beyond diagnostic accuracy

Many digital cognitive tools are initially validated in case–control designs comparing cognitively healthy participants with individuals already diagnosed with MCI or dementia. Such studies are useful for early signal detection, but they do not reproduce the uncertainty of routine practice. In primary care, the relevant distinction is often not between health and clear impairment, but between normal aging, subjective cognitive decline, depression, sleep disturbance, medication effects, sensory impairment, vascular disease, early neurodegeneration, and mixed pathology.

Implementation studies should therefore ask different questions from early validation studies. Does the tool shorten time to appropriate diagnosis? Does it increase appropriate referral rather than referral volume alone? Does it identify patients at a stage where biological confirmation or intervention is clinically meaningful? Does it perform consistently in people with low education, hearing loss, visual impairment, non-dominant language use, or low digital confidence? Does it change management? Does it reduce uncertainty for patients, caregivers, or clinicians? Does it improve access to biomarker-confirmed diagnosis, or does it widen existing gaps? (111).

Speech-based assessment illustrates both the opportunity and the challenge. Speech markers may support classification of cognitive impairment, but they may also reflect neuropsychiatric symptoms, depression, fatigue, anxiety, social withdrawal, hearing problems, or medication effects. Their interpretation is therefore strongest when embedded in a clinical model that includes mood, sleep, informant report, function, and neuropsychiatric symptoms.

For this reason, implementation studies should report pathway-level outcomes alongside accuracy: completion rate, technical failure rate, assistance required, staff time, referral yield, biomarker tests triggered, false-positive burden, clinician action, patient understanding, anxiety, and caregiver impact.

### 6.2 Patient acceptability and digital confidence

Older adults are not a homogeneous digital population. Some are experienced users of smartphones, tablets, patient portals, and video consultations; others may have limited exposure, low confidence, sensory limitations, manual dexterity problems, or anxiety about technology. Age alone is a poor proxy for digital readiness. The same digital task may therefore be experienced as convenient, neutral, stressful, or invalid depending on the person and setting.

Acceptability is shaped by perceived benefit, effort, trust, fatigue, privacy, prior technology experience, and whether results are communicated in a meaningful way. Many older adults are motivated by early detection, access to future treatments, contribution to research, and a desire to understand their cognitive health. At the same time, they may worry about technical complexity, screen fatigue, visual demands, lack of feedback, and misuse of personal data (110, 111).

These concerns should influence tool design. Digital cognitive tools intended for older adults should use short tasks, clear instructions, large readable interfaces, low-friction navigation, accessible audio and visual formats, and options for assisted administration. They should also distinguish cognitive failure from interface failure.

### 6.3 Digital equity as a condition of validity

Digital equity is often discussed as an implementation problem. In cognitive screening, it is also a validity problem. If a tool requires broadband access, smartphone ownership, comfort with tablets, language fluency, intact hearing, or familiarity with online portals, it may partly measure access and digital confidence rather than cognition (112).

The Digital Equity Screening Tool is relevant because it treats digital access and literacy as social determinants of health. Rather than assuming that patients can participate in remote digital care, it evaluates access to devices, internet connection, comfort with online health tasks, need for assistance, and language barriers. In cognitive screening, this information could determine whether a patient should complete testing remotely, in clinic, by telephone, with a caregiver, or with assisted administration (113).

Equity variables should therefore be collected before digital cognitive performance is interpreted. At minimum, studies and clinical pathways should document device access, internet stability, preferred language, sensory limitations, prior digital experience, assistance required, testing location. Women have a higher lifetime burden of AD, and sex- or gender-related differences may influence cognitive presentation, verbal performance, help-seeking behavior, caregiver roles, technology use, and confidence with digital tools (114). Validation studies should report sex/gender composition and, where sample size permits, examine subgroup calibration, particularly for speech, language, wearable, and remote-assessment models.

Voice-based tools may reduce some barriers because they do not require reading, writing, mouse use, or touchscreen fluency. However, they introduce other vulnerabilities: dialect, accent, hearing impairment, speech disorder, background noise, microphone quality, and language-model bias.

Digital equity is therefore not achieved by making a tool remote. It requires co-design, local validation, accessibility testing, language adaptation, assisted alternatives, and the option to use non-digital pathways when digital conditions are not valid.

### 6.4 Clinician trust, interpretability, and responsibility

Clinician trust depends on more than model performance. A primary-care physician, neurologist, geriatrician, psychiatrist, or neuropsychologist needs to know what the tool measures, in whom it was validated, what the output means, what uncertainty surrounds it, and what next step is recommended. A digital result that only reports “high risk” may add work rather than support decision-making.

Explainable AI can help, but explanations must be clinically meaningful. A feature-ranking plot may be useful for model developers, but clinicians need explanations that map onto recognizable constructs: reduced delayed recall, attenuated practice effect, prolonged speech pauses, reduced lexical diversity, impaired clock-drawing sequencing, abnormal gaze pattern, informant-reported decline, or functional change. Interpretability should connect the digital signal to clinical reasoning (115, 116).

However, explainability is not a substitute for validation. A transparent model can still be poorly calibrated. A high-performing model can still be biased. A clinically plausible explanation can still be wrong in an underrepresented subgroup.

Responsibility must also be explicit. Clinicians may be expected to act on algorithmic outputs generated by tools they did not select, train, validate, or supervise. This “liability sink” problem is particularly concerning when digital screening is deployed in community settings without clear escalation pathways. Governance should define responsibility at each step: developer, deploying institution, administrator, interpreting clinician, and specialist pathway. For high-consequence decisions—biomarker disclosure, AD labeling, driving advice, treatment eligibility, or exclusion from therapy—human-in-the-loop interpretation should remain mandatory.

### 6.5 Privacy, consent, and passive sensing

Because passive and semi-passive digital markers may be collected outside the traditional clinical encounter, they require stronger governance than bounded active tests. The central concern is not only privacy, but the possibility that ordinary behavior—mobility, sleep, keystrokes, voice traces, device interaction, or smart-home activity—may be reinterpreted as cognitive-risk data.

Consent should therefore be explicit, granular, revocable, and understandable. Participants should know whether raw audio, video, keystrokes, GPS traces, or only derived features are stored; whether data are used for model training; who can access the output; whether commercial partners are involved; and what happens if elevated risk is detected (117–119).

Governance should also address data minimization, cybersecurity, audit trails, model monitoring, responsibilities after abnormal results, and the right not to know. Some individuals may want digital or biological risk information even before it is clinically actionable; others may prefer to receive only results that can guide care. Digital platforms should not return uncontextualized cognitive-risk scores directly to patients without a clear disclosure pathway and human clinical oversight.

### 6.6 Regulatory status and medical device governance

Digital cognitive tools should not be assumed to be medical devices simply because they may contribute to clinical decision-making. In practice, this requires a formal regulatory assessment that considers the tool’s intended purpose, the claims made by the developer, the level of automation, the potential risk for the patient, and the extent to which the output may influence individual management. When a tool is considered medical-device software or Software as a Medical Device, the regulatory implications become clinically relevant. In Europe, this includes requirements related to clinical evaluation, CE marking, post-market monitoring, transparency, human oversight, and institutional responsibility under the Medical Device Regulation and the EU Artificial Intelligence Act. In the United States, FDA guidance on clinical decision support and AI/ML-enabled software places similar emphasis on intended use, risk classification, validation, transparency, and lifecycle monitoring. For this reason, regulatory status and governance should be reported together with technical performance, clinical validation, data protection, and workflow integration.

### 6.7 Workflow integration, reimbursement, and downstream capacity

Digital screening may reduce administration time but still increase system workload. A brief app-based or voice-based tool can screen more people, but a more sensitive tool may also increase referrals, biomarker requests, imaging demand, counseling needs, and patient anxiety. Implementation must therefore consider the full pathway, not only the time required to administer the test.

Reimbursement is part of this pathway. If digital assessment is not reimbursed, it may remain confined to research studies, private clinics, or commercial wellness platforms. If reimbursed without validation and referral criteria, it may encourage low-value testing. Health-economic evaluation should therefore report cost per person screened, cost per true positive, false-positive referral burden, biomarker test yield, time to diagnosis, number of treatment-eligible cases identified, caregiver outcomes, and equity of access.

### 6.8 Minimum reporting standards for deployment studies

The field needs reporting standards that move beyond AUC. Studies should describe the intended use case, target setting, population, digital access, test environment, device and software version, human assistance, reference standard, calibration, missingness, failure rates, subgroup performance, downstream actions, and patient impact. For AI models, reports should also include training and validation cohorts, feature types, model update plans, external validation, fairness testing, explainability methods, and data governance.

A useful principle is to report a digital cognitive screening tool both as a test and as a care process. As a test, it must show technical reliability, psychometric stability, and clinical validity. As a care process, it must show that it can be implemented safely, equitably, and usefully in the setting where it is intended to operate (120, 121).

To make digital cognitive screening studies comparable and clinically interpretable, reporting should move beyond diagnostic discrimination. Table 6 proposes a minimum reporting framework for studies that aim to support real-world deployment, particularly in primary care, remote monitoring, biomarker referral, or treatment-readiness pathways.

**Table 6:** Minimum reporting domains for digital cognitive screening studies intended for clinical translation.

Domain	Essential items to report	Reason for inclusion
Intended use	Screening, triage, monitoring, trial enrichment, biomarker referral, or treatment-readiness support; intended clinical setting and decision point	Accuracy thresholds, acceptable false-positive rates, and downstream actions depend on the clinical use case
Target population	Age, sex, education, language, ethnicity, recruitment source, symptom status, care setting, and sampling strategy	Prevents overgeneralization from selected research cohorts to primary care or community populations
Reference standard	Diagnostic criteria, adjudication process, blinding to digital results, neuropsychological assessment, functional status, informant input, and disease stage	Defines what the digital tool is actually being validated against; MCI, dementia, amyloid positivity, and AD are not interchangeable
Biological anchoring	Plasma, CSF, amyloid/tau PET, MRI markers, vascular burden, and timing between biomarker and digital assessment	Required before digital signals are interpreted as AD-relevant or used to guide biomarker referral or treatment-readiness pathways
Sampling spectrum and pretest probability	Disease prevalence, severity distribution, proportion with subjective cognitive decline, MCI, mild dementia, depression, vascular disease, sensory impairment, or low education	AUC alone does not determine clinical usefulness; predictive values depend on the tested population
Digital access and assistance	Device ownership, internet access, digital literacy, preferred language, need for caregiver/staff support, and test location	Poor performance or non-completion may reflect access barriers rather than cognition
Sensory, motor, and communication factors	Hearing, vision, speech disorder, aphasia, dysarthria, tremor, arthritis, motor impairment, and adaptations or exclusions	Speech, drawing, gaze, touchscreen, and reaction-time measures are vulnerable to non-cognitive confounding
Technical specification	Device type, operating system, software version, screen size, microphone/sensor characteristics, internet requirements, update history, and data-capture parameters	Enables reproducibility and assessment of device dependence
Task conditions	Instructions, practice trials, stimulus versions, alternate forms, test setting, time of day, background noise, lighting, interruptions, and supervision level	Remote and semi-supervised assessments are highly sensitive to environmental variability
Scoring and algorithmic model	Features used, scoring rules, thresholds, model type, training and validation samples, calibration, locked versus adaptive algorithm, and model-update plan	Determines whether the output is reproducible, interpretable, and applicable outside the development dataset
Psychometric properties	Test–retest reliability, alternate-form reliability, inter-rater reliability when relevant, practice effects, learning curves, internal consistency when appropriate, and missing-data behavior	Diagnostic accuracy is insufficient if the measure is unstable or unsuitable for longitudinal monitoring
Diagnostic performance	Sensitivity, specificity, AUC, likelihood ratios, PPV, NPV, calibration, confidence intervals, and performance at prespecified clinical thresholds	Threshold-free metrics alone do not show whether the tool supports real-world clinical decisions
Subgroup performance	Stratified performance by age, sex, education, language, ethnicity, digital access, sensory status, comorbidity, and care setting	Aggregate performance may conceal systematic misclassification in underrepresented groups
Failure modes	Non-completion, technical failure, unusable recordings, invalid trials, staff rescue, reasons for dropout, and treatment of missing data	High accuracy among completers may be misleading if real-world failure rates are high
Clinical interpretability	Clinician-facing output, uncertainty estimate, explanatory features, known confounders, contraindications, and recommended next step	A risk score without an actionable interpretation may increase uncertainty rather than improve care
Workflow consequences	Staff time, training requirements, referral rates, biomarker tests triggered, specialist appointments generated, false-positive burden, and change in management	Digital screening is a pathway intervention; efficiency at the test level may create downstream pressure
Patient and caregiver impact	Comprehension of results, acceptability, anxiety, perceived burden, caregiver involvement, preference for home versus clinic testing, and response to disclosure	Early cognitive risk information may have psychological, relational, and practical consequences
Data governance and safety	Consent model, raw versus derived data storage, data retention, secondary use, cybersecurity, audit trail, commercial access, model monitoring, and right not to know	Cognitive and behavioral data are sensitive; passive or semi-passive tools require explicit governance
Health-economic and equity endpoints	Cost per screen, cost per true positive, referral yield, cost per confirmed diagnosis, reimbursement route, regional access, and impact on inequalities	Clinical adoption should be justified by pathway value, not only by diagnostic discrimination

The table emphasizes that digital screening tools should be reported both as diagnostic instruments and as pathway interventions. AD, Alzheimer’s disease; AUC, area under the curve; CSF, cerebrospinal fluid; MCI, mild cognitive impairment; MRI, magnetic resonance imaging; NPV, negative predictive value; PET, positron emission tomography; PPV, positive predictive value.

**Figure 5:**
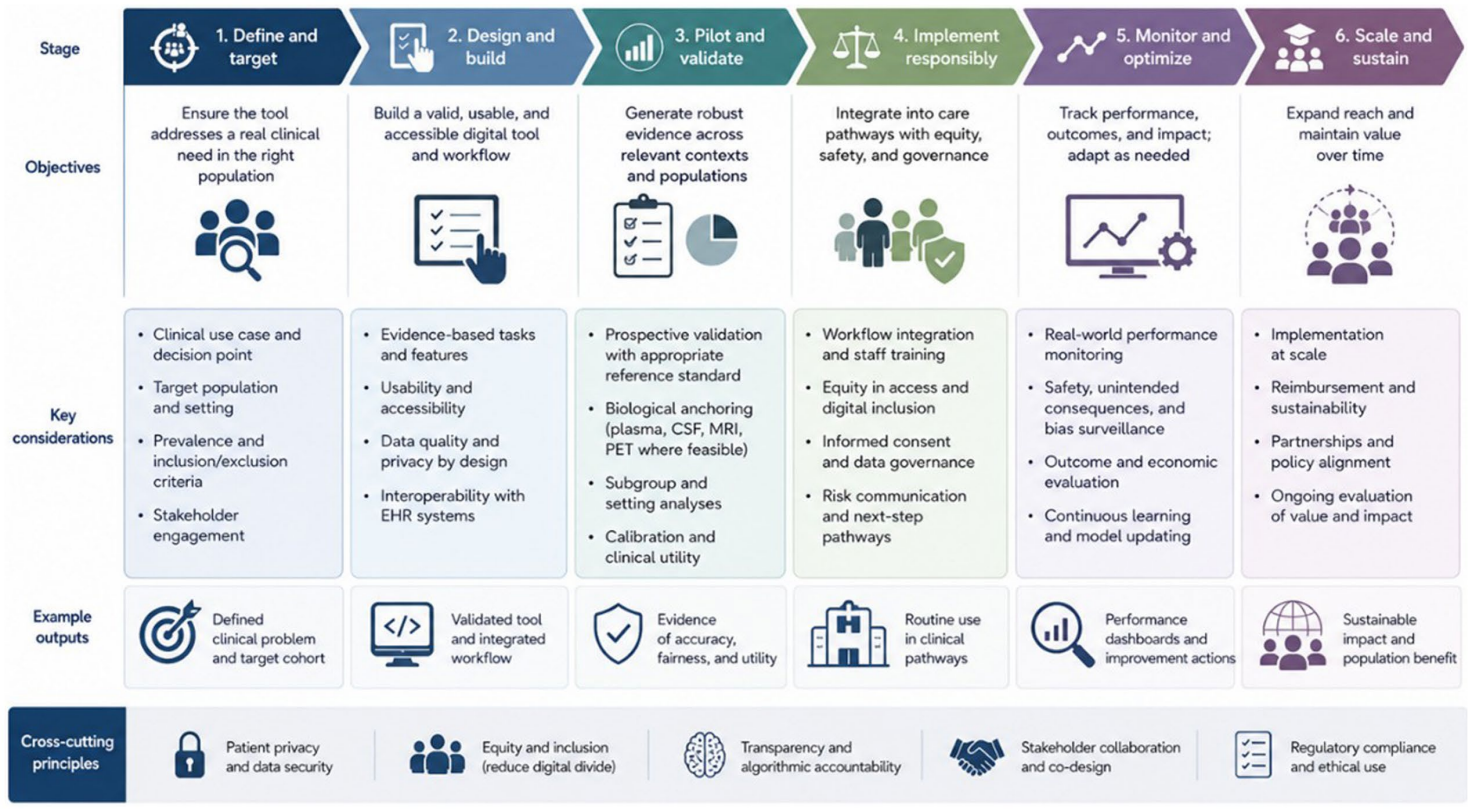
Implementation-readiness gates for digital cognitive screening. Implementation pathway from definition of clinical use case to scale-up and sustainability. The framework emphasizes validation, workflow integration, equity, governance, monitoring, and continuous learning before broad clinical deployment. CSF, cerebrospinal fluid; EHR, electronic health record; MRI, magnetic resonance imaging; PET, positron emission tomography. Created with BioRender.com.

Figure 5 summarizes these reporting requirements as implementation-readiness gates.

### 6.9 Human-centered digital neurology

The central implementation lesson is that digital cognitive screening should make care more human, not less. If digital tools reduce repetitive administration burden, standardize first-line assessment, and identify risk earlier, they may free clinicians to focus on interpretation, communication, and shared decision-making. If they deliver opaque scores, expand surveillance, or shift responsibility without support, they will undermine trust.

Narrative medicine is not opposed to digital medicine. Cognitive decline is experienced through biography: work, family roles, identity, independence, fear, care responsibilities, and future planning. A speech-derived biomarker, remote memory score, or plasma p-tau value cannot explain what memory loss means to a particular person. The clinician’s task is to translate risk into care: to decide what should be tested, what should be disclosed, what can be treated, what must be monitored, and how the patient and family can be supported.

The future of cognitive screening is likely hybrid. Brief cognitive instruments, informant reports, subjective complaints, functional scales, neuropsychiatric assessment, digital biomarkers, and biological markers should be combined in staged pathways.

## 7 Discussion: toward a hybrid, language-aware, and governed model of early cognitive detection

The evidence reviewed supports a cautious conclusion: cognitive screening should not become either a purely analog or a purely digital enterprise. Brief tests remain useful because they are inexpensive, interpretable, familiar, and already embedded in practice. Digital tools can extend this baseline by adding standardization, repeated measurement, process-level signals, and remote or more ecological assessment. Biological markers add etiological specificity and therapeutic relevance. None of these layers is sufficient alone. Early cognitive impairment detection therefore requires a staged, hybrid, clinically interpretable, biologically anchored, and governed pathway (24, 88, 92).

This matters because the target of screening has changed. The task is no longer simply to detect established dementia, but to recognize clinically meaningful change at the MCI stage, identify who may benefit from etiological confirmation, support access to disease-modifying therapies when appropriate, and avoid both underdiagnosis and overdiagnosis. A single MMSE or MoCA score cannot carry this burden, and neither can an isolated AI model based on speech, drawing, gaze, smartphone behavior, or passive sensing. The more defensible model integrates patient concern, informant-observed change, brief cognitive performance, function, neuropsychiatric symptoms, digital phenotype, and biological confirmation.

A first implication is that cognitive screening should be reframed as risk stratification plus clinical contextualization, not as a binary result. Digital tools should inherit the multidimensional logic of clinical screening rather than reduce assessment to an isolated probability score.

A second implication is that digital cognitive assessment should be judged by incremental clinical value, not technological novelty. Digitization alone is insufficient. Digitization may reduce examiner variability, but it can also preserve the limitations of the original task. Native digital signals- latency, variability, learning, speech pauses, lexical diversity, gaze, graphomotor dynamics, and real-world behavior- matter only if they change a decision: reassurance, follow-up, treatment of confounders, referral, biomarker testing, or treatment-readiness assessment. Language deserves particular emphasis. Cognitive testing is language-dependent, and performance is strongly shaped by whether assessment occurs in the patient’s dominant or native language. This issue extends beyond vocabulary. Language influences semantic fluency, naming, verbal memory, syntax, narrative organization, processing speed, test anxiety, and comprehension of instructions. In bilingual or multilingual individuals, performance may vary according to age of acquisition, language dominance, language of education, current language use, emotional salience, and sociolinguistic context. Testing in a non-dominant language can mimic impairment, whereas high verbal reserve in a dominant language may mask early decline.

This is especially relevant for AI-enabled tools. Speech biomarkers, natural-language processing, automated fluency scoring, conversational agents, and voice-recognition screeners are highly sensitive to language, dialect, accent, code-switching, and cultural narrative style. A model trained in one language community cannot be assumed to generalize to another. Translation is not enough. Local linguistic validation is required, including phonetic, lexical, semantic, syntactic, and discourse-level adaptation. This is a methodological requirement, not a cultural courtesy.

At the same time, digital tools create an opportunity for minority and minoritized languages. Traditional neuropsychological instruments are often unavailable, undernormed, or poorly adapted in smaller linguistic communities. Digital platforms may lower the cost of developing parallel tasks, collecting normative data, generating local reference distributions, and building language-specific or dialect-aware models. In multilingual regions, including those with co-official or minority languages, this could be transformative. A well-designed digital ecosystem could allow screening in the patient’s preferred or native language, reduce misclassification, and help preserve linguistic rights in healthcare. Digital cognitive screening should not reinforce the dominance of major languages; it should help build validated assessment pathways for linguistically diverse populations. This opportunity also has ethical implications: if accurate screening depends on a majority language or standardized dialect, digital innovation may widen disparities. Future studies should report language dominance, language of education, bilingualism, dialect, interpreter use, and whether testing was performed in the patient’s preferred language; for speech-based AI, subgroup performance by language and dialect should be mandatory.

A further implication is that biological anchoring is now unavoidable. Digital and analog tools can detect cognitive risk, but they cannot define AD etiology; this distinction becomes critical when validation targets shift from clinical syndromes to biomarker-defined disease (24, 25).

This definitional debate also affects how existing digital-tool validation studies should be interpreted. Many studies have validated digital measures against clinical diagnosis, MMSE or MoCA cut-offs, neuropsychological status, or syndromic MCI rather than biomarker-defined AD. If the target condition shifts toward amyloid positivity, tau positivity, biological AD, or treatment eligibility, previously reported sensitivity and specificity estimates may not transfer directly. A tool that distinguishes clinical MCI from cognitively unimpaired controls may not identify amyloid-positive or tau-positive individuals with the same accuracy. Future studies should therefore specify whether the intended target is cognitive impairment, MCI, AD dementia, amyloid positivity, tau positivity, neurodegeneration, progression risk, or treatment-readiness.

Pathway capacity remains a decisive implementation constraint: earlier detection is useful only if confirmatory diagnostics, counseling, referral pathways, and treatment-readiness infrastructure can absorb the additional demand (110, 111).

Clinician trust is another condition for safe adoption. Black-box risk scores are unlikely to be used responsibly unless they are interpretable, calibrated, and linked to clear next steps. Explainability must be clinically meaningful: a statement that classification was driven by reduced delayed recall, attenuated practice effect, prolonged speech pauses, reduced lexical diversity, abnormal drawing sequence, atypical gaze behavior, or informant-reported decline is more useful than an abstract feature-ranking plot. Yet explainability cannot substitute for validation. A transparent model can still be biased, and an accurate model can still be unsafe in the wrong population. For high-consequence decisions—biomarker disclosure, AD labeling, driving advice, treatment eligibility, or exclusion from therapy—human-in-the-loop interpretation should remain mandatory (115, 116).

Longitudinal evidence should become central: the most useful tools will be those that predict cognitive decline, functional change, biomarker progression, or treatment-relevant transitions, rather than only cross-sectional group status (23, 62). A responsible research roadmap follows from these points. Digital tools should be validated in the populations where they are intended to be used, including primary care, rural settings, low-literacy populations, multilingual communities, minority-language speakers, and older adults with sensory or motor limitations. They should be compared not only with MMSE or MoCA, but also with locally relevant brief instruments, informant measures, functional scales, neuropsychiatric instruments, and biomarker-defined outcomes. Digital screening should be embedded in predefined escalation pathways rather than deployed as direct-to-consumer risk labeling.

The goal is not to make diagnosis less human. Digital tools may quantify risk and biomarkers may clarify etiology, but clinical value emerges only when these signals are interpreted within the patient’s biography, language, culture, and goals.

In summary, digital cognitive screening should be understood as a governed extension of cognitive assessment, not as a replacement for clinical diagnosis. The field should now move from tool validation to pathway validation: demonstrating that digital screening improves real diagnostic decisions, patient outcomes, caregiver experience, health-system sustainability, and equity across languages and communities.

## 8 Conclusion

In the therapeutic era of AD, abnormal digital or brief-screening results should prompt clinical contextualization and, when appropriate, biological confirmation rather than direct etiological labeling. Plasma biomarkers, MRI, CSF, and amyloid/tau PET remain essential for etiological diagnosis, staging, and treatment-readiness decisions.

Equity is part of validity. Digital tools should be tested across education, language, culture, sex/gender, sensory and motor status, digital literacy, device access, and native-language use, particularly in bilingual and minority-language populations.

The next phase of the field should move from validating isolated tools to validating clinical pathways. Digital screening studies should demonstrate not only accuracy, but also longitudinal value, biomarker relevance, interpretability, workflow feasibility, patient understanding, caregiver impact, and health-system sustainability.

Digital cognitive screening should be used as a triage and phenotyping layer, not as a stand-alone diagnostic substitute. Its value lies in adding standardized, repeatable, and process-level signals to the clinical information already provided by brief cognitive tests, informant reports, functional assessment, neuropsychiatric symptoms, and subjective complaints.

In the therapeutic era of AD, abnormal digital or brief-screening results should prompt clinical contextualization and, when appropriate, biological confirmation, rather than direct etiological labeling. Plasma biomarkers, MRI, CSF, and amyloid/tau PET remain essential for diagnosis, staging, and treatment-readiness decisions.

Equity is part of validatiy. Digital tools should be tested across f education, language, culture, sex/gender, sensory and motor status, digital literacy, device access, and native-language use, particularly in bilingual and minority-language populations.

The field should now move from validating isolated tools to validating clinical pathways. Digital screening studies should demonstrate not only accuracy, but also longitudinal value, biomarker relevance, interpretability, workflow feasibility, patient understanding, caregiver support, and health-system sustainability.
